# *k*-mer-based GWAS reveals a candidate avirulence gene and structural variation in *Puccinia triticina* linked to gain of *Lr20* virulence

**DOI:** 10.1186/s12864-025-12230-4

**Published:** 2025-11-26

**Authors:** Ayako Tsushima, Césarée Morier-Gxoyiya, Loizos Savva, Grzegorz Czajowski, Sarah Wilderspin, Paweł Czembor, Sarah Holdgate, Diane G.O. Saunders

**Affiliations:** 1 John Innes Centre, Norwich Research Park, Norwich, UK; 2 Graduate School of Agriculture, Osaka Metropolitan University, Sakai, Japan; 3 Plant Breeding and Acclimatization Institute, National Research Institute, Radzików, Błonie, 05- 870 Poland; 4 NIAB, Cambridge, CB3 0LE UK

**Keywords:** Fungal pathogens, Wheat leaf rust, *Puccinia triticina*, Avirulence effector, Structural genomic variation, *k-*mer-based GWAS

## Abstract

**Background:**

Plant pathogens secrete effector proteins into their hosts to promote colonisation. Among these are avirulence (Avr) effectors, which can be recognised by specific host immune receptors, triggering an immune response that prevents pathogen progression. This recognition exerts strong evolutionary pressure on pathogens to alter and/or eliminate *Avr* genes to escape recognition. Consequently, understanding *Avr* gene evolution is critical for developing effective resistance deployment strategies. However, identifying and validating Avr effectors remains a significant challenge, especially for fungal plant pathogens, leading to a limited catalogue of *Avr* genes. This challenge is particularly pronounced for obligate biotrophic pathogens such as the wheat leaf (brown) rust fungus *Puccinia triticina* (*Pt*), where only two *Avr* genes have been confirmed to date.

**Results:**

In this study, we conducted a *k*-mer-based genome-wide association study (GWAS) to detect a broad spectrum of structural genetic variations — including single nucleotide polymorphisms (SNPs), insertions and deletions (indels) and copy number variations (CNVs) — that may contribute to the gain of virulence in *Pt*. Analysis of *k*-mers linked to avirulence phenotypes of *Pt* isolates across eleven leaf rust resistance (*Lr*) loci, revealed a distinct association peak on chromosome 10B corresponding to avirulence against *Lr20*. Assembly of the associated *k*-mers produced a 50 bp sequence that was located near two candidate effector genes, one of which — termed *Pt76_024702* — also displayed high levels of expression during both early and later stages of infection. Furthermore, the genomic region harbouring *Pt76_024702* exhibited large-scale deletions in certain *Pt* lineages virulent to *Lr20*, particularly those infecting durum wheat (*Triticum turgidum* ssp. *durum*).

**Conclusion:**

These findings highlight *Pt76_024702* as a compelling candidate for *AvrLr20* and demonstrate the significant potential of the presented *k-*mer-based GWAS approach to enhance the *Avr* gene catalogue. This strategy is particularly promising for complex fungal pathogens such as the notorious wheat rust pathogens where conventional approaches have previously proved challenging.

**Supplementary Information:**

The online version contains supplementary material available at 10.1186/s12864-025-12230-4.

## Background

Pathogens secrete effector proteins into plants to manipulate host processes, promote infection, nutrient acquisition, and evade host immune detection by suppressing host defence mechanisms [1]. Among these effector proteins, a subset known as avirulence (Avr) proteins can be recognised by specific plant immune receptors in certain host genotypes. This recognition triggers the hypersensitive response, resulting in localized cell death that halts pathogen proliferation [2]. Such interactions impose strong evolutionary pressure on pathogens to modify and/or eliminate *Avr* genes, enabling them to evade recognition. This dynamic process creates ‘boom-bust’ cycles, where widespread deployment of effective immune receptors in crops often leads to the emergence of new pathogen races capable of overcoming these defences [3]. A prominent example being the breakdown of the long-standing bread wheat (*Triticum aestivum*) stem rust resistance gene *Sr31* by emergence of a novel stem rust pathogen (*Puccinia graminis* f. sp. *tritici* (*Pgt*)) race termed ‘Ug99’ in Uganda in 1998, that left up to 80% of the world’s wheat varieties vulnerable to infection [4, 5]. Thus, identifying and tracking *Avr* gene evolution is critical for devising informed resistance deployment strategies.

Our understanding of Avr effectors in fungal plant pathogens has been primarily restricted to species that are genetically tractable and amenable to artificial culture. One notable example is *Fulvia fulva* (basio. *Cladosporium fulvum*) that causes tomato (*Solanum lycopersicum*) leaf mould. *F. fulva* has emerged as an important experimental model for studying plant-microbe interactions, especially since the first fungal *Avr* gene, *Avr9*, was cloned from this pathogen in 1991 [6, 7]. In contrast, the identification and validation of Avr effectors in less genetically tractable pathogens, particularly obligate biotrophs, have remained challenging. However, the onset of the genomic era revolutionised the study of complex fungal pathogens, accelerating the identification of *Avr* gene candidates. This has been especially impactful for the three wheat rust pathogens: *Pgt*, *Puccinia striiformis* f. sp. *tritici* (*Pst*) and *Puccinia triticina* (*Pt*), which cause wheat stem (black), yellow (stripe) and leaf (brown) rust, respectively [8]. These pathogens belong to the fungal kingdom (order: Pucciniales), one of the largest groups of obligate biotrophic pathogens. Recent comparative genomic studies led to identification and validation of five *Avr* genes from *Pgt* (*AvrSr13*, *AvrSr22*, *AvrSr27*, *AvrSr35* and *ArSr50*) [9–12]. However, progress in identifying *Avr* genes has been slower for other rust pathogens, where the application of similar genomic-led approaches now offers great promise for advancing *Avr* gene discovery in these species.

To date, only two *Avr* genes have been recently validated in *Pt* (*AvrLr15* and *AvrLr21*) [13, 14]. This is despite the identification of over 80 leaf rust resistance (*Lr*) loci in wheat or other grass species, such as *Aegilops*, many of which have been utilised in resistance breeding efforts, with at least a dozen of these *Lr* genes cloned [15]. However, a greater understanding of how *Pt* evades recognition – closely tied to its reproductive strategy – can guide *Avr* gene discovery. For instance, *Puccinia* species can reproduce asexually and sexually, where for *Pt* the asexual (uredinial) phase occurs on cereal hosts, while the sexual phase takes place on *Thalictrum* species [16]. Yet, in most major wheat-growing regions such as in North America and Europe, the alternative host is not considered a significant source of inoculum, and sexual reproduction of *Pt* on *Thalictrum* spp. is only found sporadically [16]. Consequently, *Pt* populations in these areas predominantly reproduce asexually, resulting in high levels of heterozygosity and linkage disequilibrium [17]. Thus, to evade recognition it is thought *Pt* relies heavily on mutation and somatic hybridization (asexual karyogamy) [18]. Accordingly, genomic-based studies to hunt for *Pt Avr* gene candidates have concentrated on identifying novel genetic combinations typical of asexual populations, generating extensive Avr protein candidate lists for *Pt* [19–21].

Focusing on genome evolution as a major potential driver of virulence in *Pt*, genome-wide association studies (GWAS) provide a powerful tool for linking genetic variation to loss of host recognition [22]. However, conventional GWAS approaches have limitations. They often miss structural variations, such as insertions/deletions (indels) and copy number variations (CNVs), which can significantly influence phenotypic diversity. These complex genetic variants are challenging to identify through simple single nucleotide polymorphism (SNP)-based associations [23], potentially underestimating their role in phenotypic traits. For instance, structural genomic variations have been implicated in escape from host recognition in several plant pathogenic fungi, including transposon-mediated gene disruption (e.g. *Verticillium dahliae*), transfer of mobile pathogenicity chromosomes (e.g. *Fusarium oxysporum*), and hybridisation (e.g., *Blumeria graminis* f. sp. *triticale*) [24–26], among others. Additionally, conventional GWAS depends on comparisons to a reference genome, which can mask associations within genomic regions absent in the single selected reference genome [27]. While long-read sequencing technologies have enabled the creation of high-quality genome assemblies and pangenome analyses, comprehensive genome data remains restricted to a handful of strains in *Pt* [18, 28, 29]. These limitations have likely hindered previous attempts to identify potential *Pt Avr* genes using GWAS-based approaches [19, 21].

Alternatively, alignment-free GWAS approaches can be used to capture a wider range of genetic variants including insertions and deletions (indels) and copy number variations (CNVs), without relying on a specific reference genome [23]. In such approaches, *k*-mers (short DNA oligomers with constant length *k*) derived directly from genomic reads are subsequently tested for association. Alignment-free GWAS has been successfully applied to various plant-associated microbes, including *Sinorhizobium meliloti* the model rhizobial species [30], *Plasmopara viticola* causing downy mildew in grape [31], and *Zymoseptoria tritici* causing septoria tritici blotch in wheat [32], among others. Notably, in *Z. tritici*, it was shown that *k*-mer-based GWAS explained a higher proportion of phenotypic variance compared to traditional SNP-based estimates across 49 phenotypic traits [32]. Thus, this alignment-free GWAS strategy holds significant promise for expanding the *Avr* gene catalogue, particularly for complex obligate biotrophic pathogens where conventional approaches have proved challenging.

In this study, we developed and applied a *k*-mer-based GWAS approach to identify candidate *Avr* genes in *Pt*. Our findings revealed that genetically similar *Pt* isolates display considerable pathotype variability, suggesting that factors beyond point mutations may contribute to the observed differences in virulence. Using this strategy, we identified a 50 bp sequence within an interspersed repeat element strongly associated with avirulence to the leaf rust resistance gene *Lr20*. Furthermore, we found a prominent *k*-mer peak linked to *Lr20* avirulence adjacent to *Pt76_024702*, a gene we propose as a strong candidate for *AvrLr20*. The gene encodes a predicted apoplastic effector and is expressed during both early and late stages of infection. Further analysis revealed complex genomic rearrangements around *Pt76_024702*, including large-scale deletions in 10 of 47 *Pt* isolates known to be virulent on *Lr20*. These findings highlight the critical role of structural variations in the adaptive evolution of *Pt* and underscore the utility of *k*-mer-based approaches in guiding future *Avr* gene identification strategies.

## Methods

### Genome sequencing of *Pt* isolates

Approximately 100 mg of dried urediniospores from 71 *Pt* isolates derived from single pustules collected in the UK and Poland (Additional file 1: Table **S1**), and multiplied through successive rounds of inoculation on the susceptible wheat varieties ‘Armada’ or ‘Michigan Amber’, were independently subjected to DNA extraction using the cetyltrimethylammonium bromide (CTAB) method as described previously [33]. DNA concentration and quality was assessed using the Qubit dsDNA HS Assay Kit (Thermo Fisher, MA, US). gDNA libraries were prepared by Genewiz (Azenta Life Sciences, MA, US) using the TruSeq DNA Library Preparation Kit (Illumina, CA, US) and the libraries were subsequently sequenced on an Illumina NovaSeq machine generating 150 bp paired-end reads.

### Assessment of genetic diversity among *Pgt*, *Pst* and *Pt* isolates

Of the 71 *Pt* genomic datasets generated herein, 69 were selected to exclude one duplicate dataset (*Pt* isolate 11–098) and one from a *Pt* isolate without available host information (Pt-14–20). Phylogenetic analysis was conducted with the remaining 69 *Pt* genomic datasets, alongside publicly available data for 58 *Pt*, 29 *Pgt* and 32 *Pst* isolates (Additional file 1: Table **S1**). Adapter and barcode trimming and quality filtering were performed on each dataset using the FASTX-Toolkit. Reads were mapped using bwa version 0.7.5 [34] to the *Pt* BBBD, Race 1 genome assembly that was repeat-masked as described previously [35]. Repetitive sequences were identified using nucmer in MUMmer version 3.23 [36] with the --maxmatch --nosimplify options, then filtered using delta-filter -l 100 -i 95. The repeat masked genome assembly was generated using BEDtools version 2.27.1 [37]. SNPs were identified using SAMtools version 0.1.19 [38], with a minimum depth of coverage of 10×, and those with allele frequencies ranging from 0.2 to 0.8 were classified as heterokaryotic. All codon positions of 342 genes with ≥ 70% breadth of coverage for at least 80% of the isolates were used to generate a maximum-likelihood phylogenetic tree using RaxML version 8.0.20 with the rapid bootstrap algorithm and 100 replicates [39]. The resulting phylogeny was visualized using iTOL version 6.1.2 [40]. To determine the degree of nucleotide diversity (π) between isolates within each of the three species (*Pt*, *Pgt* and *Pst*), SNP sites that introduced at least one nucleotide change in at least one isolate were utilised as input into DnaSP version 6 [41].

### Phylogenetic analyses of *Pt* isolates

Alongside 70 of the *Pt* genomic datasets generated herein, we sourced 140 additional publicly available genomic datasets [19, 42] (Additional file 1: Table S1). Each dataset was subjected to adapter trimming and quality filtering using BBDuk in the BBMap_38.18 package. Filtered reads were aligned to the *Pt* genome assembly (isolate Pt104; [20]) using HISAT2 2.2.1 [43] with the --no-spliced-alignment option. Average mapping depth was determined using SAMtools version 1.12 [38] with the -depth option. *Pt* isolates with >10× average mapping depth were subjected to SNP calling and phylogenetic analysis. SNP calling was performed as described previously [18] using FreeBayes version 1.3.6 (--ploidy 2) [44]. The resulting .vcf files were compressed using dgzip in HTSlib version 1.12 [45] and indexed and merged using BCFtools version 1.17 [46]. SNPs were filtered using vcffilter of VCFlib version 1.0.3 [47] with the parameter -f ‘QUAL >20 & QUAL/AO >10 & SAF >0 & SAR >0 & RPR >1 & RPL >1 & AC >0’. Biallelic SNPs were selected using VCFtools (-min-alleles 2 -max-alleles 2 -max-missing 0.9 -maf 0.05 --recode) [48] and converted to multiple sequence alignments in PHYLIP format using vcf2phylip version 2.8 [49]. The phylogenetic tree was constructed using IQ-TREE2 version 2.3.3 [50] with an ultrafast bootstrap approximation (UFBoot) option (-st DNA -nt AUTO -B 1000 -alrt 1000 -bnni) [51] and visualised in iTOL version 6.9 [40] with the *Pt* isolate ISR850 collected on *Aegilops speltoides* [42] used as an outgroup.

### Virulence profiling of *Pt* isolates

Urediniospores were harvested from 66 *Pt* isolates and multiplied by successive rounds of inoculation on the susceptible wheat varieties ‘Armada’ or ‘Michigan Amber’. For each round of inoculation, wheat plants were grown in controlled conditions (16-h light/8-h dark and 18 °C/12°C cycles). Urediniospores were resuspended in NovecTM 7100 engineered fluid (Sigma-Aldrich, UK) and used for spray inoculation of three to five seedlings (biological replicates) once they reached the two leaf stage following described procedures [52]. *Pt* isolates were used for inoculation of wheat lines, which harbour the following leaf rust resistance (*Lr*) genes: *Lr1*, *Lr10*, *Lr11*, *Lr14a*, *Lr17*, *Lr20*, *Lr21*, *Lr24*, *Lr26*, *Lr28*, and *Lr30.* At approximately 10–12 days post-inoculation, *Pt* infection types were assessed on the first seedling leaf using a 0–4 infection type scale according to [53], where scores of 0–2.3 indicate avirulent reactions, scores of 2.4–2.6 borderline reactions and scores of 2.7–4.0 virulent reactions. Polish isolates were classified as 0 or 1. 0, reflecting avirulent and virulent reactions, respectively.

### Reference-free *k*-mer-based GWAS

To assess *Pt* genomic datasets for potential contamination we selected 34 datasets with below a 70% mapping rate and 10× mapping depth against the *Pt* reference genome (isolate Pt104; [20]). Datasets were analysed using SendSketch in the BBMap_38.94 package, sampling 50% of the first 5 million reads from the unmapped read subsets and using the minkeycount = 2 option. The unmapped reads were extracted using SAMtools version 1.12 [38] with the -view option. Primary contaminant organisms were defined as those with the highest score in the output file. Reference-free *k*-mer-based GWAS was conducted on 128 *Pt* genomic datasets where pathotype data was available using HAWK 1.6.0 Beta [54], with a minimum *k*-mer count of 3 and excluding pathotypes with intermediate scores. Quantile–quantile (Q-Q) plots were generated using the R package qqman version 0.1.8 [55]. Principal component analyses were performed using Eigenstrat [56] and visualised using the R package ggplot2 version 3.4.4. *k*-mers with significant association were assembled using ABySS version 2.0.2 with the options ‘-k25 -c0 -e0’ [57]. All *k*-mers were also mapped to the *Pt* genome assemblies (isolate Pt76) for haplotype A and B independently including unplaced contigs [18] using Bowtie version 2.4.1 [58]. Resulting .bam files were used to generate Manhattan plots using the R package qqman version 0.1.8 [55]. Genomic regions 25 Kb up- and down-stream from the gene containing the assembled *k*-mer sequences was extracted using SeqKit v2.3.0 [59]. Protein sequences for genes within these regions were assessed for secretion signals using SignalP version 6.0 with default settings [60] and analysed using EffectorP version 3.0 to identify potential cytoplasmic or apoplastic effectors [61].

### Genome sequence alignments

The genome sequence alignments were performed using nucmer with the –maxmatch option and visualised using mummerplot in MUMmer4 version 4.0.0rc1 [36]. We extracted the 25 Kb up- and down-stream genomic region from *Pt76_024699* on Pt76 chromosome 10B using SeqKit v2.3.0 [59]. Similarly, for 19NSW04 chromosome 10B, we extracted the 25 Kb up- and down-stream genomic regions from the *Pt76_024699* homolog, *19NSW04_024885*. As the homologous sequence to *Pt76_024699* was separated into two genes on 20QLD87 chromosome 10D, we extracted the 25 Kb up-stream region from *20QLD87_025914* and the 25 Kb down-stream region from *20QLD87_025915*. Pt76 chromosome 10A, 19NSW04 chromosome 10 C and 20QLD87 chromosome 10 C were devoid of *Pt76_024699* homologs. Thus, we extracted the genomic sequence 37 Kb up-stream and 16 Kb down-stream from *Pt76_023579*, *19NSW04_023783* and *20QLD87_024737*, which are homologs of the *AvrLr20* candidate gene, *Pt76_024702* on Pt76 chromosome 10B.

Following adapter and barcode trimming and quality filtering, genomic reads were mapped using HISAT2 version 2.1.0 with the --no-spliced-alignment --no-softclip --mp 6,2 --score-min L,0.0,−0.12 options to the *Pt* Pt76 B genome without unplaced contigs [43] to exclude ambiguously mapped reads. Resulting .sam files were converted into .bam files using SAMtools version 1.12 [38] that were further reformatted into .bigwig files using bamCoverage version 3.5.1 in deepTools with default settings [62]. The log-scaled mapping depth graphs were visualised using Integrated Genomic Viewer version 2.15.2 [63]. The genomic regions commonly absent in the 10 *Pt* isolates collected on *Triticum turgidum* ssp. *durum* were defined as follows: Genomic regions with low mapping rates (< 1/2 of the average mapping depth over the 25 Kb up/downstream genomic region) were identified using SAMtools version 1.13 [38] and common overlaps identified and regions merged if they are closer than 150 bp using BEDTools version 2.26.0 [37]. Among the resulting regions, five regions longer than 1 Kb were considered as genomic regions commonly absent.

### RNAseq and differential expression analyses

A total of 100 *Pt*-infected wheat field samples were subjected to RNA extraction using the RNeasy Plant Mini Kit (QIAGEN, Venlo, Netherlands) and quantity and quality assessed using an Agilent 2100 Bioanalyzer (Agilent Technologies, CA, US). Complementary DNA libraries were prepared using the TruSeq RNA Sample Preparation Kit (Illumina, CA, US) and sequenced on the Illumina Hiseq 2500 machine by Genewiz (Azenta Life Sciences, MA, US), generating 150 bp paired-end reads. Publicly available RNA-seq data for Pt76 [64] was subjected to adaptor and barcode trimming, and quality filtering using BBDuk in the BBMap_38.18 package. Filtered reads were aligned to the *Pt* Pt76 dikayotic genome including unplaced contigs using the HISAT2 version 2.2.1 with the --max-intronlen 3000 option as described previously [64]. The resulting .sam files were converted into .bam files and the mapping depth graph visualised as described above. Read counts for each gene were generated using featureCounts bundled in subread 2.0.6 using the settings -p -M --fraction -t exon -g gene_id. Differentially expressed genes (DEGs) were defined using the R package DEseq2 version 1.38.3 with the thresholds padj < 10e-6 and log2FoldChange >4. The upset plot was generated using the R package UpsetR version 1.4.0. Transcripts per million (TPM) counts were generated using StringTie version 2.2.1 [65] with default settings and boxplots visualised using the R package ggplot2 version 3.4.4.

### Yeast signal sequence trap assay

To confirm the secretory function of the putative *Pt76_024702* secretion signal, we used the yeast signal sequence trap method, with the pSUC2T7M13ORI (pSUC2) vector that contains a truncated *SUC2* invertase gene [66]. A 102 bp fragment including the predicted Pt76_024702 secretion signal (Pt76_024702^1–32^) was synthesised and cloned into the pSUC2 vector [67] in frame with the invertase gene by Azenta Life Sciences (Burlington, Massachusetts, USA). An invertase secretion-deficient *Saccharomyces cerevisiae* strain (YTK12) was transformed with pSUC2-Pt76_024702^1–34^ or the pSUC2-PexRD8^1–24^ positive control [67] using the lithium acetate method [68]. Yeast was plated on YPDA media (1% yeast extract, 2% peptone, 2% glucose, 0.003% adenine hemisulfate and 2% agar) and incubated at 30 °C for 2–3 days, with positive clones confirmed by Sanger sequencing. Positive clones were grown on single dropout media (SD) devoid of tryptophan (0.67% yeast N base without amino acids, 0.075% tryptophan dropout supplement, 2% sucrose, 0.1% glucose, and 2% agar) plates or restrictive media with raffinose as the sole carbon source (YPRAA media: 1% yeast extract, 2% peptone, 2% raffinose, 2 µg/ml antimycin A and 2% agar) to assay for invertase secretion. Plates were incubated at 30 °C for 3–4 days. Invertase enzymatic activity was analysed as described previously [67], through assessment of reduction of 2,3,5-triphenyltetrazolium chloride (TTC: Sigma-Aldrich, Merck KGaA, Darmstadt, German) to red coloured insoluble triphenylformazan. Untransformed YTK12 and the same strain transformed with the empty pSUC2 vector, were used as negative controls.

### AlphaFold protein structure prediction

The modelled monomeric structure of Pt76_024702 was obtained from the AlphaFold Protein Structure Database (UniprotID: A0A180GT65 [69]), which provides predicted protein structures using the AlphaFold2 algorithm [70]. The predicted aligned error (PAE) and per-residue confidence scores (predicted Local Distance Difference Test, pLDDT) were retrieved to evaluate the reliability of the model. The retrieved structure was visualized using Mol* version 4.16 [71].

## Results

### *Pt* has low genetic diversity among isolates when compared to *Pgt* and *Pst* isolates

**Fig. 1:**
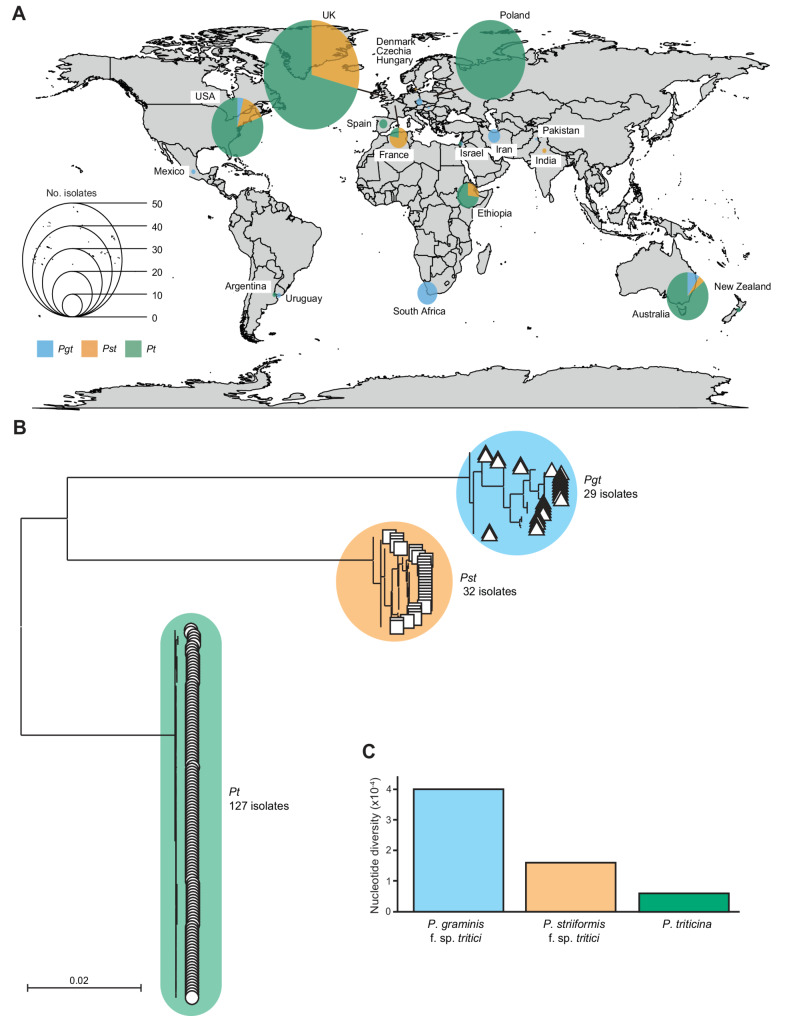
Genetic diversity between isolates is lower for *Pt* than *Pgt* or *Pst*. **A** A total of 188 genomic datasets representing 127 *Pt*, 29 *Pgt* and 32 *Pst* isolates were sourced from a diverse array of countries. **B** Phylogenetic analysis grouped *Puccinia* isolates into three clades reflective of the three distinct species (*Pt*, *Pgt* and *Pst*), with *Pt* isolates exhibiting the least genetic differentiation, as reflected in short branch lengths. Phylogenetic analysis was performed using all codon positions of 342 gene models (475,969 sites) that were conserved among at least 80% of all *Puccinia* isolates, with a maximum likelihood approach. **C** The degree of genetic diversity between the geographically diverse *Puccinia* isolates was substantially lower among *Pt* isolates than *Pgt* or *Pst*. Nucleotide diversity (π) was determined using single nucleotide polymorphisms (SNPs) identified in the 342 conserved genes

To assess the level of global genetic diversity among *Pt*,* Pgt* and *Pst* isolates, we sequenced 71 *Pt* isolates collected in the UK and Poland between 2006 and 2016 and compiled publicly available genomic data from geographically diverse isolates of each wheat rust pathogen. This dataset included isolates sourced from 10 countries each for *Pt* and *Pgt* and 7 countries for *Pst* (Fig. 1a and Additional file 1: Table S1). Reads from the 29 *Pgt*, 32 *Pst* and 127 *Pt* isolates were independently aligned to the *Pt* reference genome (isolate BBBD, Race 1 [35]). On average, *Pgt* isolates showed 17.31% alignment (4.77 ± S.D.), *Pst* isolates 13.12% (4.75 ± S.D.) and *Pt* isolates 75.87% (14.59 ± S.D.) (Additional file 1: Table S2). To assess genetic similarity across these isolates, we performed phylogenetic analysis of all *Pt*,* Pgt* and *Pst* isolates (475,969 sites; Fig. 1b). The resulting phylogeny revealed distinct clades for each species, with *Pt* isolates exhibiting the least genetic differentiation, as reflected in short branch lengths. This was further supported by the much lower nucleotide diversity (π) identified among *Pt* isolates (6.0 × 10^−5^), compared to *Pgt* (4.0 × 10^−4^) and *Pst* (1.6 × 10^−4^) (Fig. 1c). Overall, these findings align with previous reports of reduced genetic differentiation among geographically diverse *Pt* isolates relative to *Pgt* and *Pst* [17, 72].

### Genetically similar *Pt* isolates can display considerable variability in their virulence profiles

**Fig. 2:**
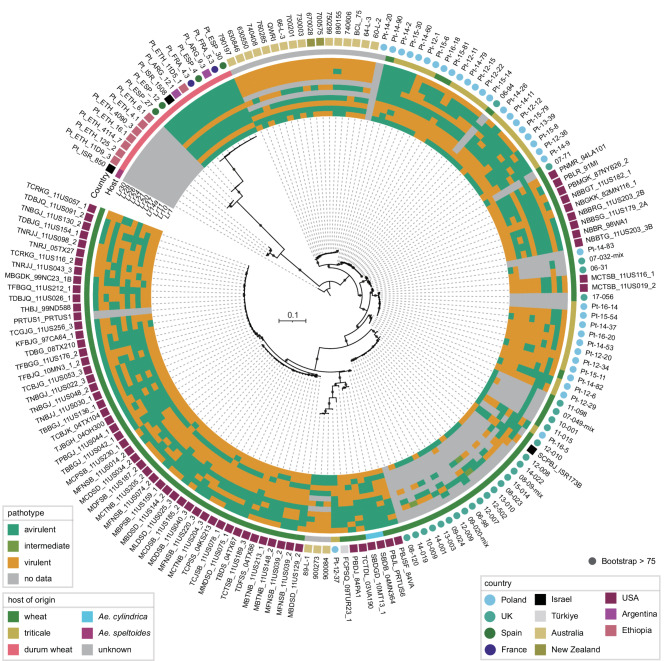
*Pt* isolates with low genetic differentiation display considerable variation in virulence profiles. *Pt* isolates generally clustered into clades based on their geographic or host origins, despite substantial pathotype diversity. Phylogenetic analysis was performed that incorporated 171 *Pt* genomic datasets, using biallelic single nucleotide polymorphisms (SNPs) (89,733 sites) and a maximum likelihood approach. *Pt* isolate ISR850 collected on *Aegilops speltoides* was designated as an outgroup. Pathotype diversity is indicated where known, with reactions for up to 11 leaf rust resistance gene (*Lr*) loci

To further investigate the genetic diversity among *Pt* isolates, we collated 83 additional publicly available *Pt* genomic datasets [19, 42] and aligned all 210 *Pt* datasets to the *Pt* reference genome (isolate Pt104 [20]) (Additional file 1: Table S1). Datasets with an average mapping depth of less than ×10 were removed from subsequent analysis, resulting in the removal of 39 datasets (Additional file 2: Fig. S1 and Additional file 1: Table S3). Phylogenetic analysis of the remaining 171 *Pt* isolates (89,733 sites) (Fig. 2) revealed that *Pt* isolates generally clustered into clades based on their geographic or host origins, consistent with previous findings [18, 42]. For example, 17 of 20 isolates from Australia and New Zealand formed a single clade, and all 17 *Pt* isolates collected on durum wheat (*T. turgidum* ssp. *durum*) grouped together in the phylogeny. However, the Polish *Pt* isolates exhibited a less clear pattern, with those collected from wheat and triticale (a hybrid between wheat and rye) distributed across two main clades, while three *Pt* isolates (Pt-14–83, Pt-16-5 and Pt-12–37) appeared as singletons (Fig. 2).

To determine if the low genotypic diversity among *Pt* isolates was also reflected in pathotype variation, we pathotyped 66 of the 71 newly sequenced *Pt* isolates using 11 near-isogenic wheat lines carrying different leaf rust resistance (*Lr*) genes (*Lr1*, *Lr10*, *Lr11*, *Lr14a*, *Lr17*, *Lr20*, *Lr21*, *Lr24*, *Lr26*, *Lr28*, and *Lr30*) (Additional file 1: Table S4). We also gathered publicly available pathology data for 131 additional *Pt* isolates from previous studies [19, 42], which revealed substantial pathotype diversity among the 171 *Pt* isolates (Additional file 1: Table S5). Among the 11 *Lr* loci tested, *Lr21* was the most effective, with only 25.0% of *Pt* isolates displaying virulence, while *Lr10* was the least effective, with 76.8% of *Pt* isolates displaying virulence (Fig. 2).

Pathotype variation generally correlated with the genetic structure observed in the phylogeny. For instance, all *Pt* isolates collected on durum wheat (*T. turgidum* ssp. *durum*) clustered together and showed a distinctive pathotype, characterised by virulence on *Lr10* and *Lr20* (Fig. 2). Polish *Pt* isolates were distributed across two large clades reflecting two primary pathotype groups: (i) avirulence on *Lr10*, *Lr11*, *Lr14a*, and (ii) avirulence on 10 of the 11 *Lr* loci, except *Lr28*. However, other genetically similar *Pt* isolates differed in their pathotypes. For example, most *Pt* isolates collected from Australia and New Zealand formed a single clade but varied in their virulence to *Lr1* and *Lr20*. Similarly, *Pt* isolates from the UK displayed variable virulence on *Lr1*, *Lr20* and *Lr26* (Fig.2). Overall, these analyses reveal high levels of pathotype diversity among *Pt* isolates, which are not entirely captured by their groupings in the SNP-based phylogenetic analysis.

### Reference-free GWAS identified *k*-mers significantly associated with nine *Lr* loci

**Fig. 3:**
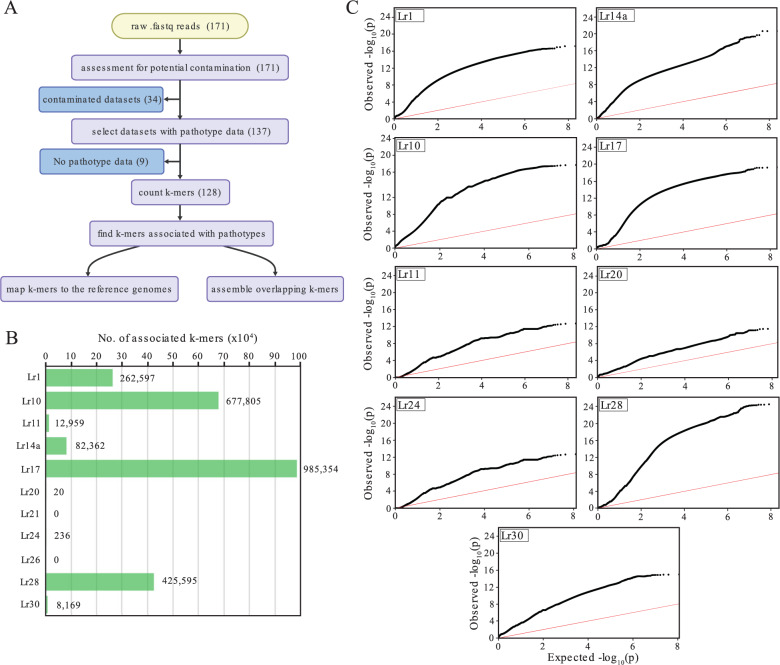
*k-*mer-based GWAS identified sequences significantly associated with the avirulence phenotype against nine *Lr *loci. **A** Schematic illustrating the steps of the *k*-mer-based GWAS analysis. Following quality filtering of 171 genomic *Pt* datasets, 34 were excluded due to potential contamination, and 9 due to a lack of available pathotype data. *k-*mers were assessed in each of the remaining 128 datasets and those different between avirulent and virulent *Pt* isolates were highlighted. Detected *k-*mers were mapped to the reference genome and/or assembled into contigs. **B** Association analysis identified *k-*mers linked to avirulence for nine out of 11 leaf rust resistance gene (*Lr*) loci. **C** Quantile-quantile (Q-Q) plots of the -log_10_(*p*-values) displayed curved distributions for all nine *Lr* loci, indicating a much greater association in the genome than expected under the null. Red line indicates expected distribution

Given that certain *Pt* isolates exhibited limited SNP variation but divergent pathotypes, we sought to investigate whether structural variations (SVs) could contribute to the observed pathotype diversity. To address this, we employed a *k*-mer-based GWAS approach, broadening our genotypic analysis to include SVs such as insertions and deletions (indels) and copy number variations (CNVs) alongside SNPs (Fig. 3a). Prior to conducting GWAS, we screened the 171 *Pt* genomic datasets to exclude any with potential contamination that might skew the results. This analysis identified 34 *Pt* isolates with low alignment rates to the *Pt* reference genome (less than 70% of reads aligned, mean ± 56.39 and S.D.±12.08), suggesting possible contamination (Additional file 3: Fig. S2 and Additional file 1: Table S3). To investigate further, we conducted sequence similarity searches on 2.5 million randomly extracted unmapped reads from each of these 34 *Pt* datasets. This revealed high levels of reads similar to other organisms including *Frankliniella occidentalis* (thrips), *Rhopalosiphum maidis* (aphids) and several symbiotic bacteria (Additional file 1: Table S6). Consequently, these 34 *Pt* datasets were removed from further analysis. From the remaining 137 *Pt* datasets, 128 with available pathotype profiles were selected for *k*-mer-based association mapping (Additional file 1: Table S1). Using *k*-mers of length 31, we identified ~ 1.2 billion *k*-mers across the 128 *Pt* datasets. The association analysis highlighted significant *k*-mers associated with avirulence against nine out of 11 *Lr* loci, with the number of associated *k*-mers ranging from 20 to 985,354 (Fig. 3b and Additional file 1: Table S7).

During the reference-free *k-*mer-based GWAS analyses we noted that the Quantile-Quantile (QQ) plots of the -log_10_(*p*-values) displayed substantial deviations from the expected distribution (Fig. 3c), with the curved distributions indicating a much greater association in the genome than expected under the null, likely due to population stratification. To explore potential adjustments to account for these underlying structural differences we focused on *Lr20* where the Q-Q plot was closest to the expected distribution, and altered the number of principal components identified by Eigenstrat [56] (Additional file 4: Fig. S3 and Additional file 5: Fig. S4) and/or removed *Pt* isolates collected from durum wheat (*T. turgidum* ssp. *durum*), which were genetically distinct in the phylogenetic tree (Fig. 2). Although these adjustments reduced the *p-*value inflation, deviations from the expected distribution in the resulting Q-Q plots were still evident (Additional file 6: Fig. S5). Thus, we maintained default settings, all *Pt* isolates and included the first two principal components identified by Eigenstrat in all future analyses.

### *k*-mer-based GWAS analyses identified *AvrLr20* gene candidates

**Fig. 4:**
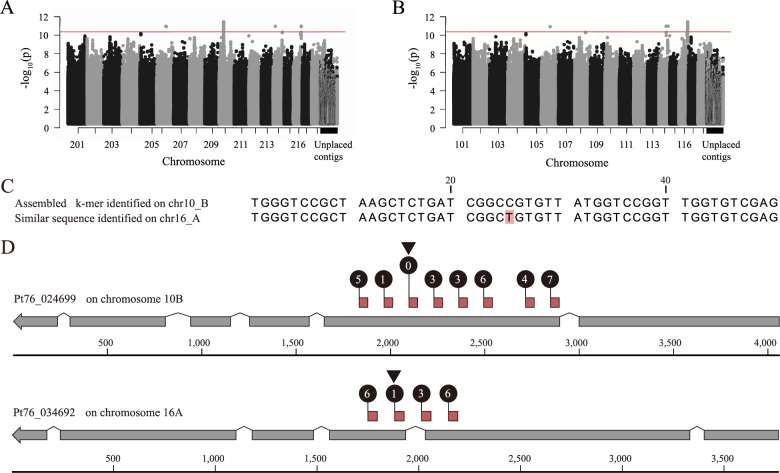
*AvrLr20 *gene candidates were found in the genomic regions harbouring the significantly associated *k*-mers. **A**, **B**) Manhattan plots for the avirulent phenotype against the leaf rust resistance gene (*Lr*) loci, *Lr20* in the B or A haplotype genomes of *Pt* isolate Pt76, respectively. Horizontal red line indicates the Bonferroni significance threshold (α < 0.05). **C** The 50-bp sequence assembled from *k-*mers linked to *Lr20* virulence, was identified within *Pt76_024699* on chromosome 10B and *Pt76_034692* on chromosome 16 A. *Pt76_034692*, differed from the assembled 50 bp sequence by a single nucleotide, highlighted with pink shading. **D** Tandem duplications of the assembled 50 bp *k*-mer sequence were evident in *Pt76_024699* and *Pt76_034692*. Grey arrows, gene models; pink squares, location of the 50 bp sequence; numbers in black circles, number of nucleotide variations from the assembled *k*-mer sequence

To further investigate *Pt k*-mers associated with avirulence phenotypes against the nine *Lr* loci, we aligned the *k*-mers to the B haplotype genome of *Pt* isolate Pt76 [64]. The average alignment rate of *k*-mers was 56.07% (13.78 ± S.D.), with uniquely aligned *k*-mers accounting for 25.53% (6.74 ± S.D.) and multi-aligned *k*-mers making up 29.53% (S.D. ± 7.26) (Additional file 1: Table S8). The high proportion of multi-mapping *k*-mers likely reflects the prevalence of repeat sequences, which cover more than 60% of the *Pt* genome [64]. Using aligned *k*-mers, Manhattan plots were generated for the nine *Lr* loci (*Lr1*, *Lr10*, *Lr11*, *Lr14a*, *Lr17*, *Lr20*, *Lr24*, *Lr28* and *Lr30*). These plots illustrated numerous *k*-mers below the significance threshold after Bonferroni correction for all *Lr* loci except *Lr20*, which displayed one pronounced peak on chromosome 10B (Fig. 4a and Additional file 7: Fig. S6). To assess the robustness of the peak on chromosome 10B, we examined the region when altering the number of principal components. The peak on chromosome 10B associated with *Lr20* virulence was generally sustained, except when using a maximum of ten principal components (Additional file 1: Table S9 and Additional file8: Fig S7), indicating the peak was largely maintained even when substantially varying the GWAS parameters.

Since the reference *Pt* isolate (Pt76) is avirulent on *Lr20* [64], it likely harbours the corresponding avirulence effector allele (*AvrLr20*) in one or both of its dikaryotic genomes. The *k*-mer peak on chromosome 10B could therefore correspond to the *AvrLr20* allele. To determine whether a similar peak exists in the alternate genome, the *k*-mers were aligned to the A haplotype genome of Pt76. The mapping rates were comparable to those obtained with the B haplotype genome (uniquely aligned *k*-mers: 23.19%; multi-aligned *k*-mers: 28.32%) (Additional file 1: Table S8). A single peak was identified on chromosome 16A (Fig. 4b), suggesting the locus could be heterozygous between the two genomes in *Pt* isolate Pt76.

**Fig. 5:**
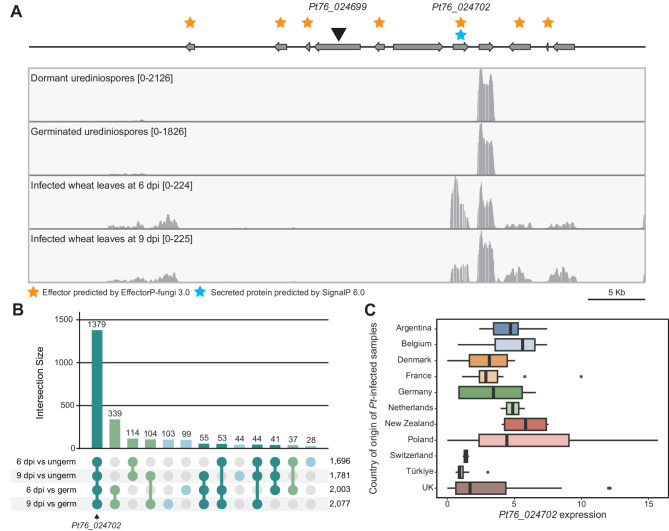
*AvrLr20 *candidate *Pt76_024702 *is upregulated during wheat infection. **A** *Pt76_024702* was highly expressed at 6 and 9 days-post inoculation (dpi) in publicly available RNA-seq data. Schematic displays the 25 Kb region up- and down-stream of *Pt76_024699* on chromosome 10B. Black triangle, location of the *k-*mer-assembled 50-bp sequence; numbers in brackets, minimum and maximum depths of coverage. **B** *Pt76_024702* is among 1,379 *Pt* genes upregulated *in planta* out of the 26,892 genes analysed. Intersection sizes are illustrated for *Pt* genes identified as differentially upregulated *in planta* when compared to datasets from dormant and germinated *Pt* urediniospores. **C ***Pt76_024702* was highly expressed in field-collected *Pt*-infected plant samples collected across 11 countries (100 RNA-seq datasets analysed). Transcripts per million (TPM) values for *Pt76_024702* illustrated with bars representing median values and boxes signifying the upper (Q3) and lower (Q1) quartiles, and whiskers are located at 1.5 the interquartile range

To further investigate the sequence associated with the *Lr20*-linked *k*-mers, we assembled the detected *k*-mers, generating a 50 bp sequence (5’-CTCGACACCAACCGGACCATAACACGGCCGATCAGAGCTTAGCGGACCCA-3’). Sequence similarity searches identified an exact match for this 50 bp sequence on chromosome 10B within the gene *Pt76_024699* and a match on chromosome 16A within the gene *Pt76_034692*, which differed from the assembled 50 bp sequence by a single nucleotide (Fig. 4c). Both genes contained tandem duplications of the *k*-mer sequence, with 8 or 4 repeats and up to 7 or 6 base variations for *Pt76_024699* and *Pt76_034692*, respectively (Fig. 4d). To assess whether *Pt76_024699* and/or *Pt76_034692* encode putative effector proteins, we analysed their predicted protein products for secretion signals and used machine learning to predict effector potential [60, 61]. Neither gene encoded a signal peptide or was predicted as a cytoplasmic or apoplastic effector using current available methods. Therefore, we expanded the search to include genes within 25 Kb up- and down-stream of *Pt76_024699* and *Pt76_034692*. This analysis identified two genes encoding proteins with predicted secretion signals and known effector characteristics. These include *Pt76_024702*, located 9,975 bp down-stream of the 50 bp *k*-mer sequence on chromosome 10B (Fig. 5a), predicted as an apoplastic effector, and *Pt76_034691*, located 2,025 bp up-stream of the *k*-mer sequence on chromosome 16A, predicted as a cytoplasmic effector (Additional file 9: Fig. S8 and Additional file 1: Table S10). Given their proximity to the *k*-mers associated with avirulence against *Lr20*, these genes (*Pt76_024702* and *Pt76_034691*) are strong candidates for *AvrLr20*.

### The *AvrLr20 *candidate gene is upregulated in expression during early and late stages of infection and encodes a secretory protein

To further evaluate *Pt76_024702* and *Pt76_034692* as candidates for *AvrLr20*, we analysed their expression during infection using publicly available RNA-seq data for *Pt* isolate Pt76 [64]. RNA-seq reads from dormant urediniospores, germinated urediniospores, and *Pt*-infected wheat leaves at 6- and 9-days post inoculation (dpi) were aligned to the Pt76 reference genome (Additional file 1: Table S11). The results showed that *Pt76_024702* is among 1,379 genes upregulated *in planta* out of the 26,892 genes analysed, whereas *Pt76_034692* is not significantly up- or down-regulated *in planta* as it displayed almost no detectable transcripts across all four developmental stages (Fig. 5a and 5b and Additional file 1: Table S12). To investigate *Pt76_024702* and *Pt76_034692* expression during later stages of infection, when pustules are abundant, we analysed 100 RNA-seq datasets derived from field-collected *Pt*-infected plant samples across 11 countries. The datasets were aligned to the Pt76 reference genome, yielding an average alignment rate of 20.60% (S.D. ± 12.30%) (Additional file 1: Table S13). Expression of *Pt76_024702* varied widely among *Pt*-infected wheat samples, with the greatest degree of in-country expression variation across *Pt* isolates collected in Poland (Fig. 5c and Additional file 1: Table S14). Whereas *Pt76_034692* expression was consistently low as the expression value of this gene was less than 1.0 in 92 out of the 100 *Pt* isolates tested. (Additional file 1: Table S14 and Additional file 9: Fig. S8b). These findings suggest that *Pt76_024702* is expressed during both early and later stages of infection.

**Fig. 6:**
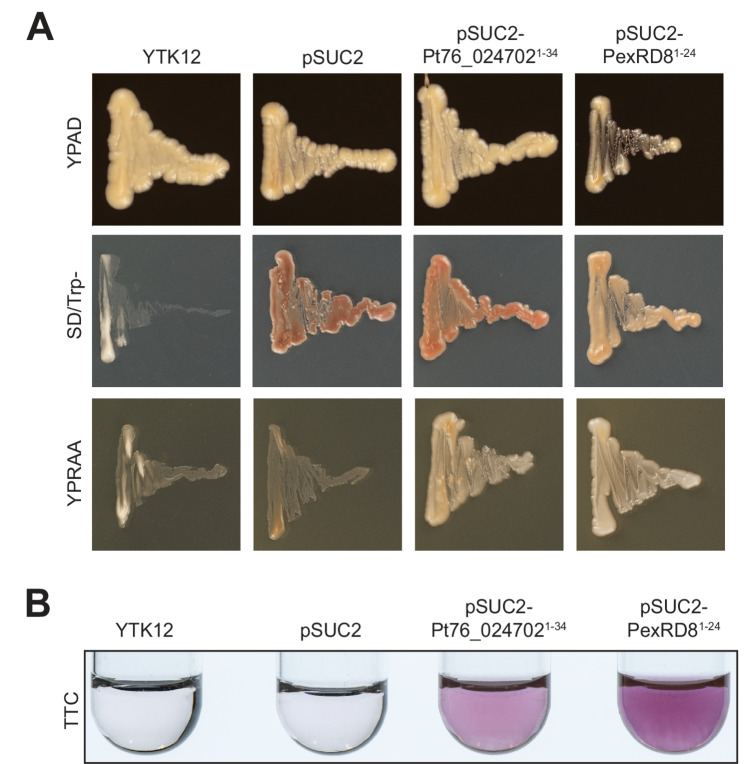
Pt76_024702 contains a functional signal peptide. **A** The *Saccharomyces cerevisiae* YTK12 strain transformed with pSUC2-Pt76_024702^1–34^ grew well on SD/Trp- medium, indicating successful transformation and on restrictive YPRAA medium confirming the secretory function of the Pt76_024702 signal peptide. The YTK12 strain, which lacks endogenous invertase secretion, was transformed with pSUC2 (negative control), pSUC2-Pt76_024702^1–34^, or pSUC2-PexRD8^1–20^ (positive control). **B** Invertase secretion from YTK12 transformed with pSUC2-Pt76_024702^1–34^ or pSUC2-PexRD8^1–20^ was confirmed by enzymatic activity, with successful reduction of triphenyltetrazolium chloride (TTC) to red-coloured insoluble triphenylformazan (TF). No colour change was observed in untransformed YTK12 or cells transformed with empty pSUC2 vector, indicating absence of invertase secretion

To confirm whether *Pt76_024702* encodes a *Pt* protein with potential *in planta* function, we assessed the predicted signal peptide region for secretory function. The yeast strain YTK12 that is invertase secretion-deficient was transformed independently with either pSUC2, pSUC2-Pt76_024702^1–34^ or the positive control pSUC2-PexRD8^1–24^ [67]. After 2 days of incubation, all YTK12 strains grew on SD/Trp- medium, confirming successful transformation (Fig. 6a). On restrictive YPRA media containing raffinose as the sole carbon source, only strains harbouring pSUC2-Pt76_024702^1–34^ or pSUC2-PexRD8^1–24^ displayed robust growth, while those with the empty pSUC2 vector or no construct lacked growth (Fig. 6a). These results were further supported by enzymatic activity assays: only YTK12 strains carrying pSUC2-Pt76_024702^1–34^ or pSUC2-PexRD8^1–24^ reduced triphenyltetrazolium chloride (TTC) to insoluble red triphenylformazan (TF), indicating active invertase secretion (Fig. 6b). These results demonstrate that the N-terminus of Pt76_024702 contains a functional signal peptide, supporting a potential *in planta* function.

### The *AvrLr20* gene candidate is in close proximity to structural genomic variations

**Fig. 7:**
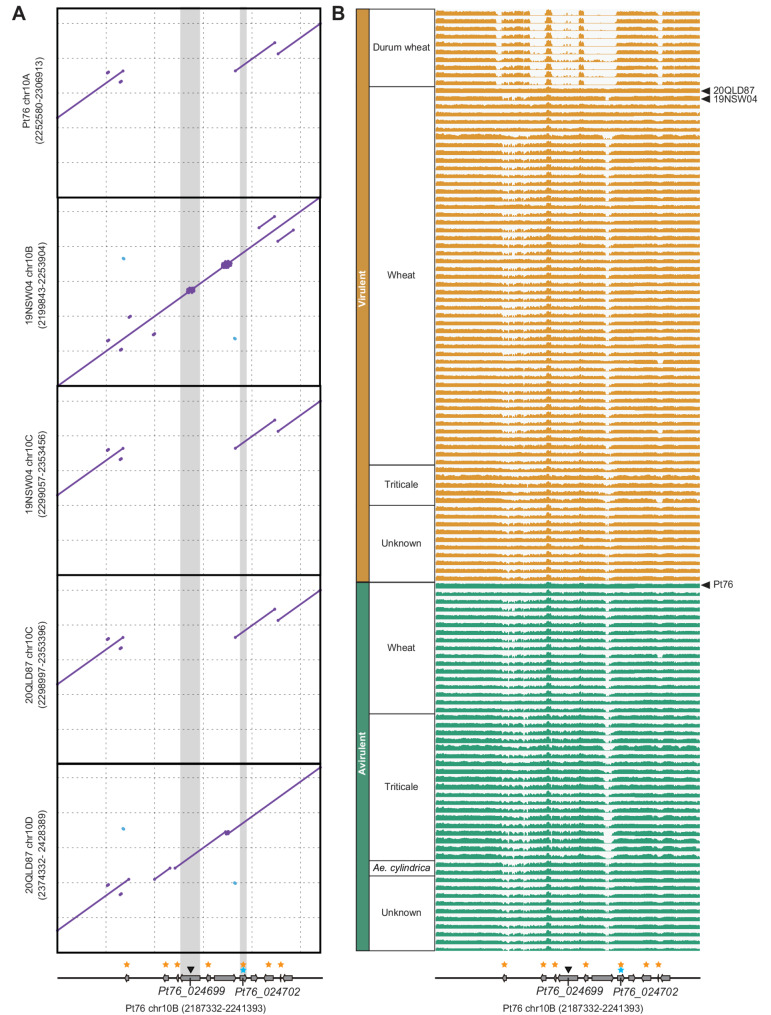
*AvrLr20 *gene candidate *Pt76_024702 *is within proximity of structural genomic variation. **A** Sequence alignments revealed presence/absence heterozygosity of the *k*-mer region associated with leaf rust resistance gene (*Lr*) *Lr20* virulence up-stream of *Pt76_024699*. Genomic sequence alignments of the 25 Kb up- and down-stream region flanking *Pt76_024699* on chromosome 10B (isolate Pt76) and the corresponding regions from chromosome 10A (isolate Pt76), chromosome 10C (isolate 19NSW04) and chromosome 10D (isolate 20QLD87). Numbers, start and end positions of each genomic region; purple and blue dots, forward and reverse matches, respectively; grey shading, positions of *Pt76_024699* and *Pt76_024702*; black triangle, significantly associated *k-*mers; yellow stars, genes encoding effectors predicted by EffectorP-fungi version 3.0; blue star, gene encoding putative signal peptide. **B** Ten of the 47 *Pt* isolates virulent on *Lr20* had large-scale deletions evident in the region that contained the significantly associated *k*-mer sequences (black triangle). Graphs indicate the mapping depth of 119 *Pt* isolates, alongside *Pt* isolates Pt76, 19NSW04 and 20QLD87 using 50-bp bins in the 25 Kb up- and down-stream region flanking *Pt76_024699* on chromosome 10B (isolate Pt76). y-axis represents log-scale, adjusted to the maximum value for each *Pt* isolate

To investigate potential associations between *k*-mers significantly linked to *Lr20* avirulence and structural variations, we conducted genomic sequence alignments. These alignments compared the 25 kb up- and down-stream regions flanking *Pt76_024699* on chromosome 10B with the corresponding genomic region on chromosome 10A in Pt76. This analysis successfully identified a gene with identical sequence to *Pt76_024702* on chromosome 10A, termed *Pt76_023579* (Additional file 9: Fig. S8). Further examination of the gene model for *Pt76_023579* initially suggested a potential 66-bp deletion in the N-terminus when compared to *Pt76_024702*. However, this was due to a shorter gene model being called for *Pt76_023579*, which when corrected was identical in length and sequence to *Pt76_024702* (Additional file 10: Fig. S9). Furthermore, we noted that *Pt76_024702* is adjacent to a 23,100 bp genomic region enriched with significantly associated *k*-mers on chromosome 10B, which is absent from the region up-stream of *Pt76_023579* on chromosome 10A in Pt76 (Fig. 7a). To further examine the genomic context, we analysed two recently sequenced *Pt* isolates virulent against *Lr20* and where phased genomes were available (*Pt* isolates 19NSW04 and 20QLD87 [18]). Sequence alignments revealed presence/absence heterozygosity around the *k*-mer-associated regions between the haploid genomes, similar to Pt76 (Fig. 7a). Homologs of *Pt76_024702* were present in both *Pt* isolates and encoded identical proteins to Pt76_024702 (Additional file 10: Fig. S9), but upstream regions of these homologs displayed deletions or inversions (Fig. 7a), suggesting complex genomic rearrangements around the *k*-mers associated with *Lr20* avirulence.

To investigate further the genomic variation on chromosome 10 between *Pt* isolates, we mapped the genomic reads for 119 *Pt* isolates used in *k*-mer-based GWAS, alongside Pt76, 19NSW04 and 20QLD87, to the Pt76 haplotype B genome assembly. This revealed that 10 of the 47 *Pt* isolates known to be virulent on *Lr20* had large-scale deletions (totalling 17,607 bp) evident in the region that contained the significantly associated *k*-mer sequences (Fig. 7b and Additional file 1: Table S15). We noted that all 10 *Pt* isolates with large-scale deletions were collected from durum wheat (*T. turgidum* ssp. *durum*), where *Lr20* virulence is more prevalent [73]. To determine whether this deletion is widely conserved among durum wheat *Pt* isolates, we examined 11 additional durum wheat-derived *Pt* isolates that had been excluded from the *k*-mer-based GWAS analyses due to low mapping rate/depth and/or lack of pathotype data (Additional file 1: Table S16). Alignment of the genomic reads of these 11 *Pt* isolates to the Pt76 haplotype B genome assembly, indicated that 7 of the 11 *Pt* isolates also harbour a similar large-scale deletion in the region (Additional file 11: Fig. S10). Additionally, while *Pt* isolates 19NSW04 and 20QLD87, which are virulent on *Lr20*, had reads covering most of the region, whole-genome alignments showed it was only present in one of the two dikaryotic genomes (Fig. 7a). This highlights the limitation of short-read mapping in distinguishing heterozygous null deletions. These findings suggest that the genomic region harbouring the *AvrLr20* candidate gene is associated with large-scale deletions in *Pt* isolates virulent against *Lr20*, particularly those found on durum wheat.

### Pt76_024702 is highly conserved in sequence between *Pt* isolates and unique to a subset of *Puccina* species

To assess the level of conservation in the *Pt76_024702* sequence among *Pt* isolates, we analysed its sequence across the 119 *Pt* isolates used for *k*-mer-based GWAS, as well as Pt76, 19NSW04 and 20QLD87. This analysis revealed a high degree of conservation, with 17 point mutations in intron and UTR regions identified in at least one of the 122 *Pt* isolates and only one non-synonymous mutation (S205T) observed in *Pt* isolate Pt-16-5 that is avirulent for *Lr20* (Additional file 1: Table. S17). Examination of the predicted structure of Pt76_024702 using AlphaFold [70] illustrated that it was intrinsically disordered, containing extensive flexible regions (Additional file 12: Fig. S11), similar to other filamentous pathogen effectors [74]. We also investigated the conservation of *Pt76_024702* among other fungal species through sequence similarity searches against the NCBI non-redundant protein sequence database. This analysis showed that *Pt76_024702* appears to be unique to *Puccinia* species, with homologs detected only in *Pgt*, *Pst*, and *Puccinia coronata* f. sp. *avenae* (BlastP, E-value ≤ 2 × 10^−11^) (Additional file 1: Table S18). Together, this indicates that *Pt76_024702* is highly conserved among *Pt* isolates and appears to be specific to *Puccinia* species. 

## Discussion

To date, the identification of fungal *Avr* genes has primarily hinged on comparative genomic approaches and the identification of allelic variation linked to virulence gains [75]. Accordingly, among the wheat rust pathogens comparative genomic approaches were central to the identification of the first three *Avr* genes from *Pgt* (*AvrSr50*, *AvrSr35*, and *AvrSr27*), with virulence gains largely associated with *Avr* gene disruption and/or allelic variation [9–11]. However, unlike *Pgt* that displays high levels of SNP-based divergence between isolates, *Pt* isolates display the least SNP-based genetic variation among the wheat rust pathogens, despite considerable pathotype variability. This lower level of SNP-based genetic variation likely impeded the identification of *Avr* genes for *Pt* as other forms of genetic variation could be playing a more prominent role in driving virulence gains in *Pt* that are not captured in current comparative genomic studies. To explore this, we employed a reference-free *k-*mer-based GWAS approach to search for structural genomic variations in *Pt* that could be associated with gain of virulence for eleven *Lr* loci. Among those tested, we identified an association between *Lr20* avirulence and a 50 bp *k*-mer sequence among a subset of *Pt* isolates. Detailed analysis of the genomic region harbouring *k*-mers associated with *Pt Lr20* avirulence, revealed the region had undergone structural rearrangements in certain *Pt* lineages; 10 of 47 *Pt* isolates virulent to *Lr20* had large scale deletions, whereas none of the 72 *Pt* isolates avirulent to *Lr20* exhibited deletions in this region. Furthermore, all 10 *Pt* isolates with *Lr20* virulence were collected exclusively on *T. turgidum* ssp. *durum*, where *Pt* isolates are known to be genetically divergent from those found on *Triticum aestivum* [17]. Thus, the divergent genome structure of these *Pt* isolates from *T. turgidum* ssp. *durum* could underpin the high level of *Lr20* virulence in this lineage [73].Further examination of the genomic region associated with the 50 bp *k*-mer sequence led to identification of two potential *AvrLr20* candidates (*Pt76_024702* and* Pt76_034691*) within close proximity to the *Lr20* linked *k*-mers. These candidates were located within 10 Kb of the 50 bp *k*-mer sequence and displayed all typical features of effector proteins. In addition, we found that *Pt76_024702*, but not *Pt76_034692*, was upregulated in expression during both early and later stages of infection, further supporting a role for *Pt76_024702* in supporting successful *Pt* infection. However, both genes were absent from the previous list of 20 candidates suggested for *AvrLr20* through SNP-based association analyses [19]. We surmise this is likely due to the high level of *Pt76_024702* sequence conservation between *Pt* isolates with differing *Lr20* virulence. Despite this sequence conservation, we did find structural variations such as deletion, repeat sequence amplifications and indels were seemingly common in the genomic region proceeding *Pt76_024702*, which could reflect alterations in transcriptional regulators of *Pt76_024702*. Similarly, the confirmed *Pt Avr* gene *AvrLr21* also displayed complete sequence-conservation between *Lr21* avirulent and virulent isolates. However, the *Pt AvrLr21* gene was expressed at twice the level in *Lr21*-avirulent isolates compared to *Lr21*-virulent isolates, allowing the latter to evade* Lr21* recognition [14]. In future, it would be advantageous to generate RNAseq data to explore the expression levels of *Pt76_024702* between *Lr20* avirulent and virulent isolates to determine if expression polymorphisms are a dominant strategy used by *Pt* to potentially evade host recognition.

As a compelling candidate for *AvrLr20*, the next step is to confirm the avirulence function of *Pt76_024702*. However, this remains a significant challenge, primarily due to the difficulty of functionally validating *Avr* genes in obligate biotrophic pathogens like the wheat rust fungi, which are notoriously resistant to genetic transformation [76]. Cloning the corresponding *R*-gene would significantly expand the possibilities for validating the proposed Avr function of Pt76_024702. *Lr20* is known to be located on wheat chromosome 7AL and genetically associated with *Pm1a* and *Sr15* resistance effective against the wheat powdery mildew and stem rust pathogens, *Blumeria graminis* f. sp. *tritici* (*Bgt*) and *Pgt* [77]. The successful cloning of *Pm1a* demonstrates how modern genomic-based techniques can overcome earlier challenges linked to limited genetic recombination [77], offering a promising pathway to expedite the cloning of *Lr20* and other previously elusive *R* genes. Once *Lr20* is cloned, validation of Avr function for Pt76_024702 could be pursued either through pairwise transient co-expression with *Lr20* in surrogate systems like *Nicotiana benthamiana* or by co-delivery of Pt76_024702 and Lr20 into wheat protoplasts [12].

Despite the presented *k-*mer-based GWAS strategy holding significant promise for expanding the *Avr* gene catalogue, its application to complex obligate biotrophic pathogens remains limited, likely due to persistent challenges. For instance, although we successfully identified *k-*mers associated with *Lr20* virulence, we failed to identify sequences associated with *Pt* virulence for the other 10 *Lr* genes considered. This could in part be due to an array of inherent technical challenges, including the inconsistency in *Pt* pathotyping results between available datasets, bias in *Pt* isolates selected and/or inclusion of *Lr* genes that encode non-nucleotide-binding and leucine-rich repeat (NLR) proteins. For instance, *Lr34* and *Lr67* encode transporters, where gain of virulence may not follow the typical gene-for-gene concept [78, 79]. As the analysis of reference pathogen collections expands – especially through large-scale genome sequencing projects – it will enhance the availability of high-quality data required for future *k-*mer-based GWAS studies.

## Conclusions

Fungal phytopathogens employ an array of mechanisms to modify *Avr* effectors to evade host recognition. To capture a wide range of genetic variants, including SNPs, indels and CNVs, that could be associated with gain of virulence in *Pt*, we applied a *k-*mer-based GWAS approach. This led to the identification of *k-*mers specifically associated with *Lr20* virulence that were in close proximity to an *Avr* effector gene candidate (*Pt76_024702*) that was also highly expressed during infection. Furthermore, we revealed that the genomic region containing this gene has undergone complex genomic rearrangements in *Pt* isolates displaying *Lr20* virulence, which are usually difficult to detect using traditional SNP-based approaches. Thus, our work highlights the potential of *k*-mer GWAS to capture genomic variations more comprehensively and understand virulence mechanisms of obligate biotrophic phytopathogens with highly complex genomic structures.

## Supplementary Information

Supplementary Material 1.

Supplementary Material 2.

Supplementary Material 3.

Supplementary Material 4.

Supplementary Material 5.

Supplementary Material 6

Supplementary Material 7.

Supplementary Material 8.

Supplementary Material 9.

Supplementary Material 10.

Supplementary Material 11.

Supplementary Material 12.

## Data Availability

All custom computer code has been deposited on GitHub (https://github.com/SaundersLab/LR_kmer_GWAS) and the genomic data that supports the findings of this study has been deposited in the European Nucleotide Archive (accession number PRJEB84390).
