# Supplementary figures and images for: k-mer-based GWAS reveals a candidate avirulence gene and structural variation in Puccinia triticina linked to gain of Lr20 virulence
Source: BMC Genomics. 2025 Nov 26;26:1076. doi: 10.1186/s12864-025-12230-4 (PMC12659103; doi:10.1186/s12864-025-12230-4)

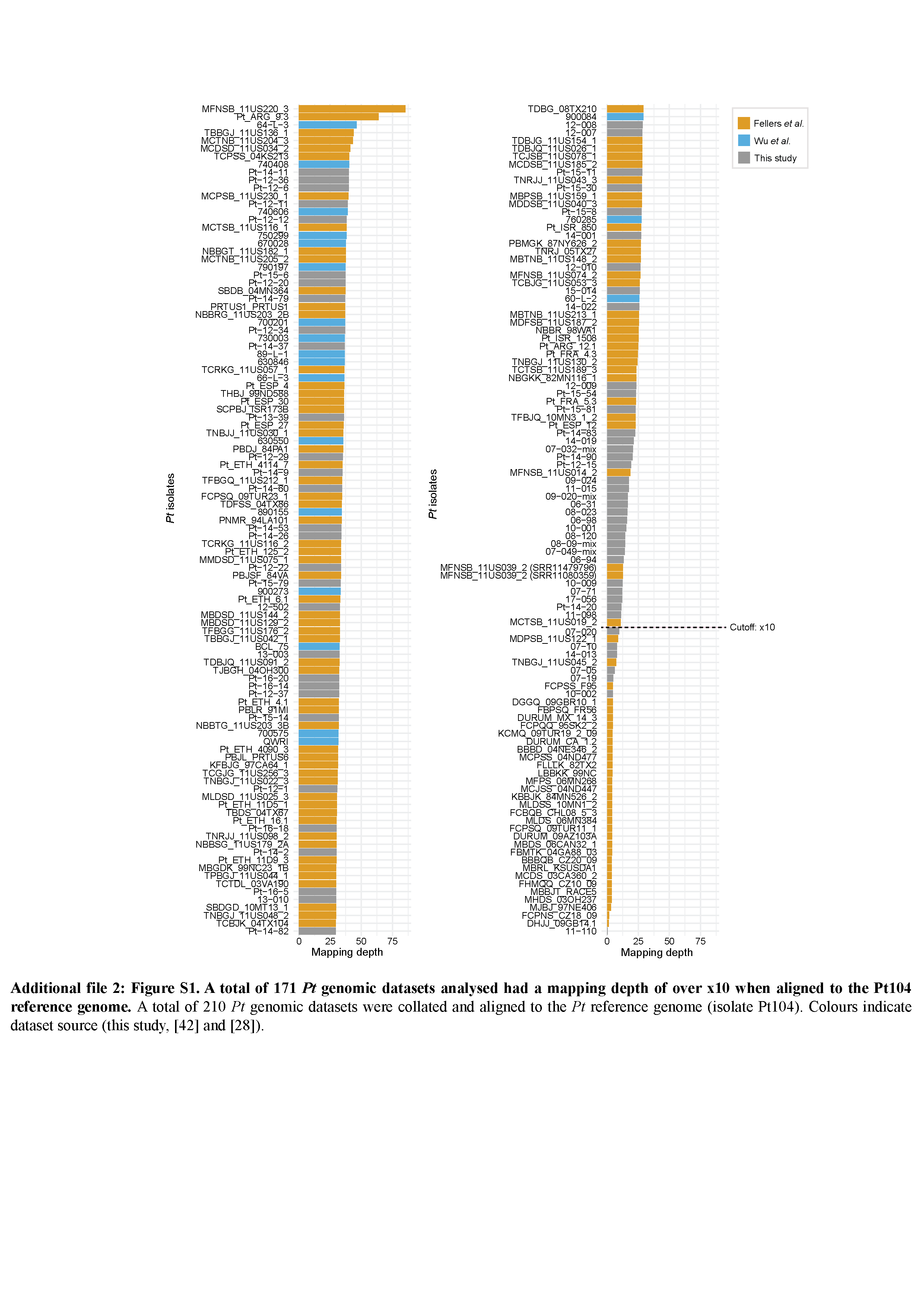

Supplement: Supplementary file 2 — Supplementary Material 2. [file 12864_2025_12230_MOESM2_ESM.tif]

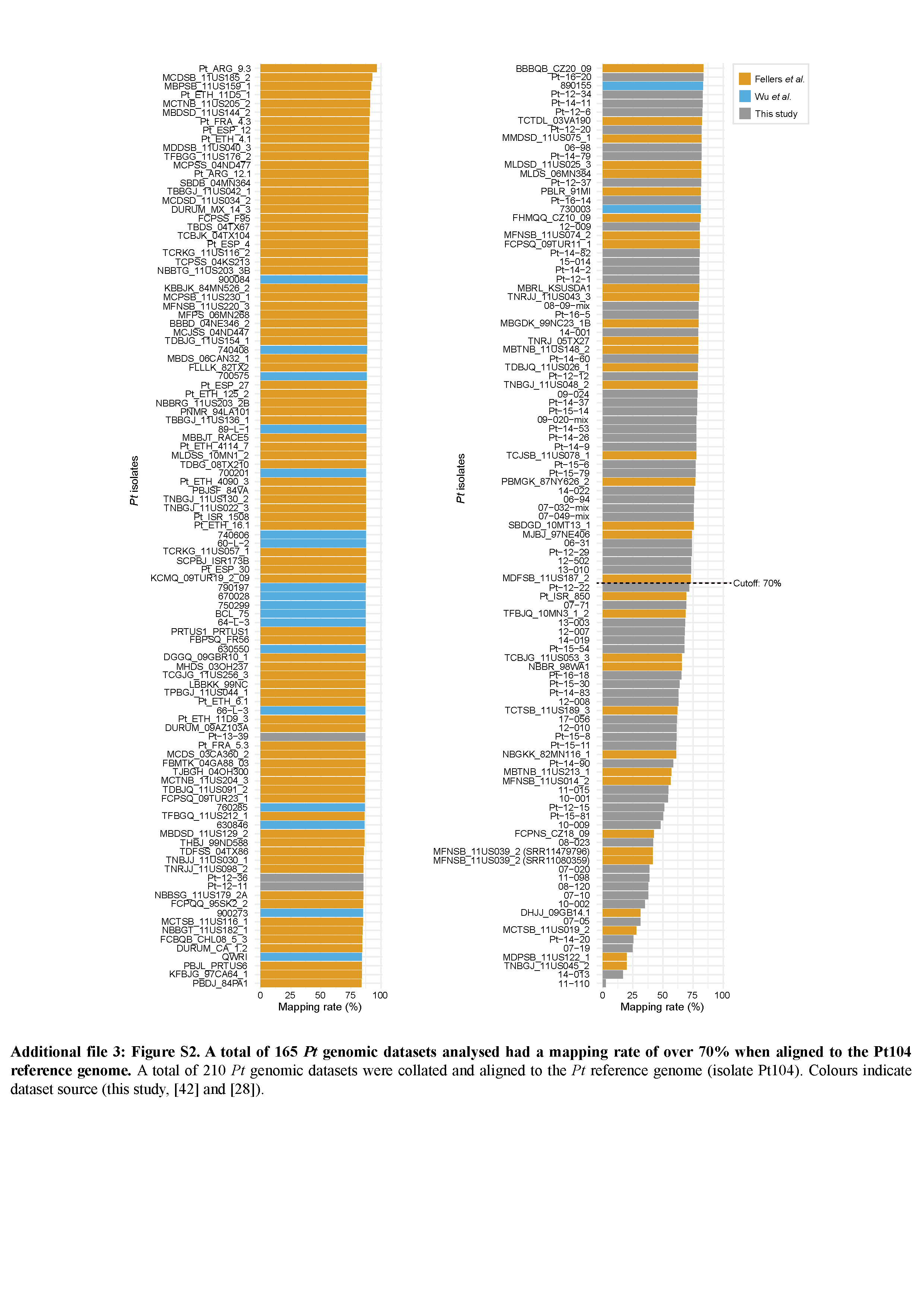

Supplement: Supplementary file 3 — Supplementary Material 3. [file 12864_2025_12230_MOESM3_ESM.tif]

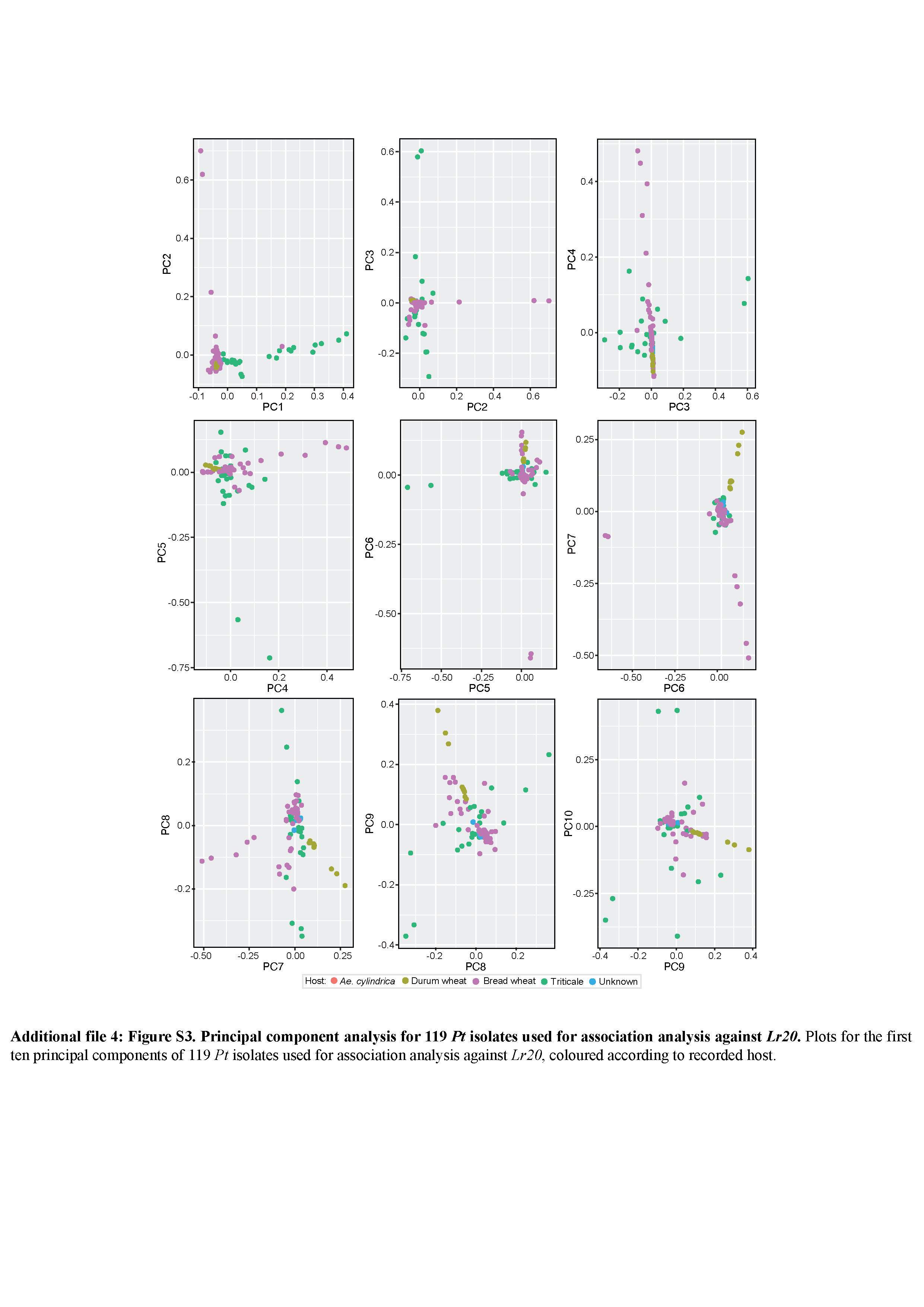

Supplement: Supplementary file 4 — Supplementary Material 4. [file 12864_2025_12230_MOESM4_ESM.tif]

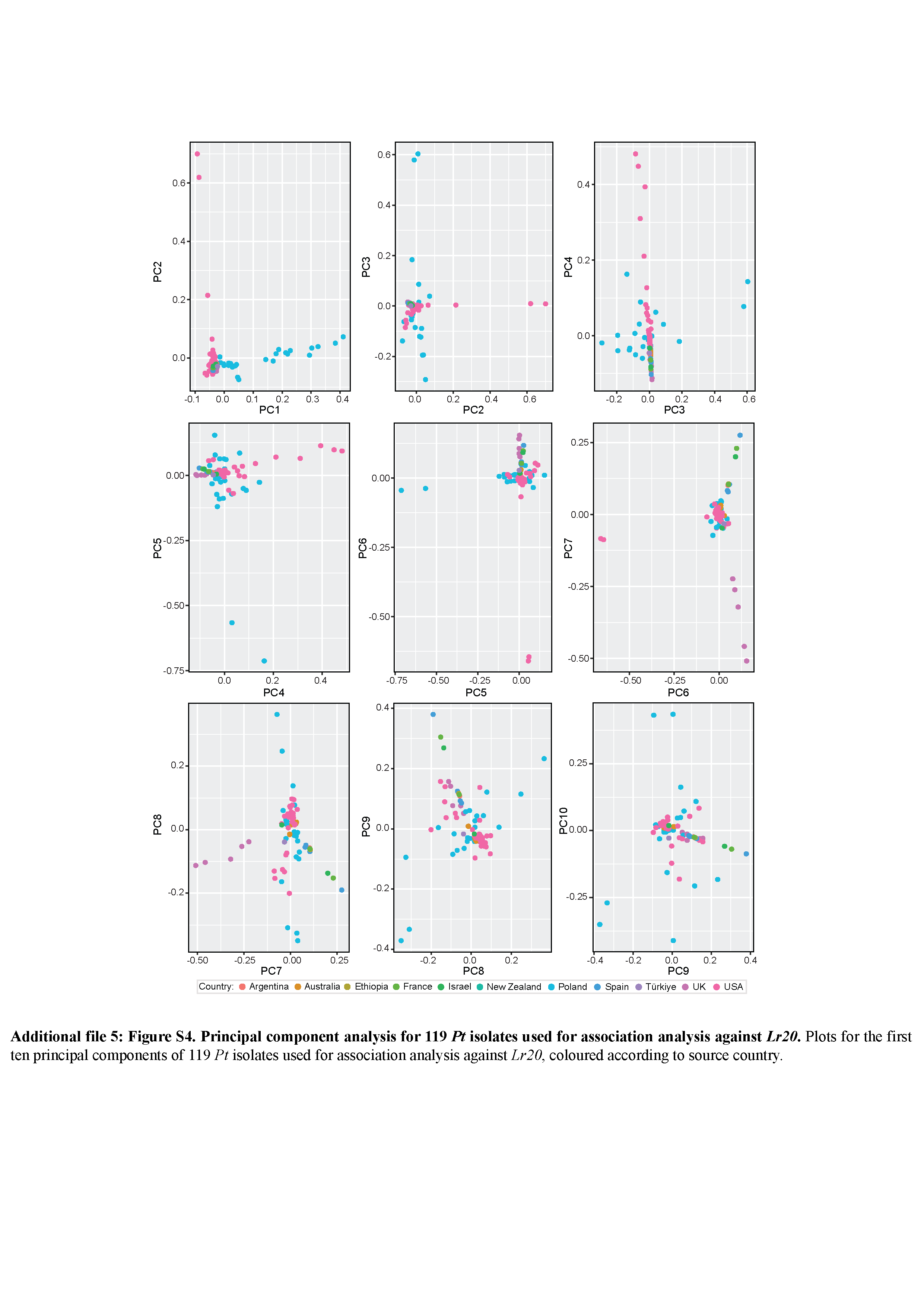

Supplement: Supplementary file 5 — Supplementary Material 5. [file 12864_2025_12230_MOESM5_ESM.tif]

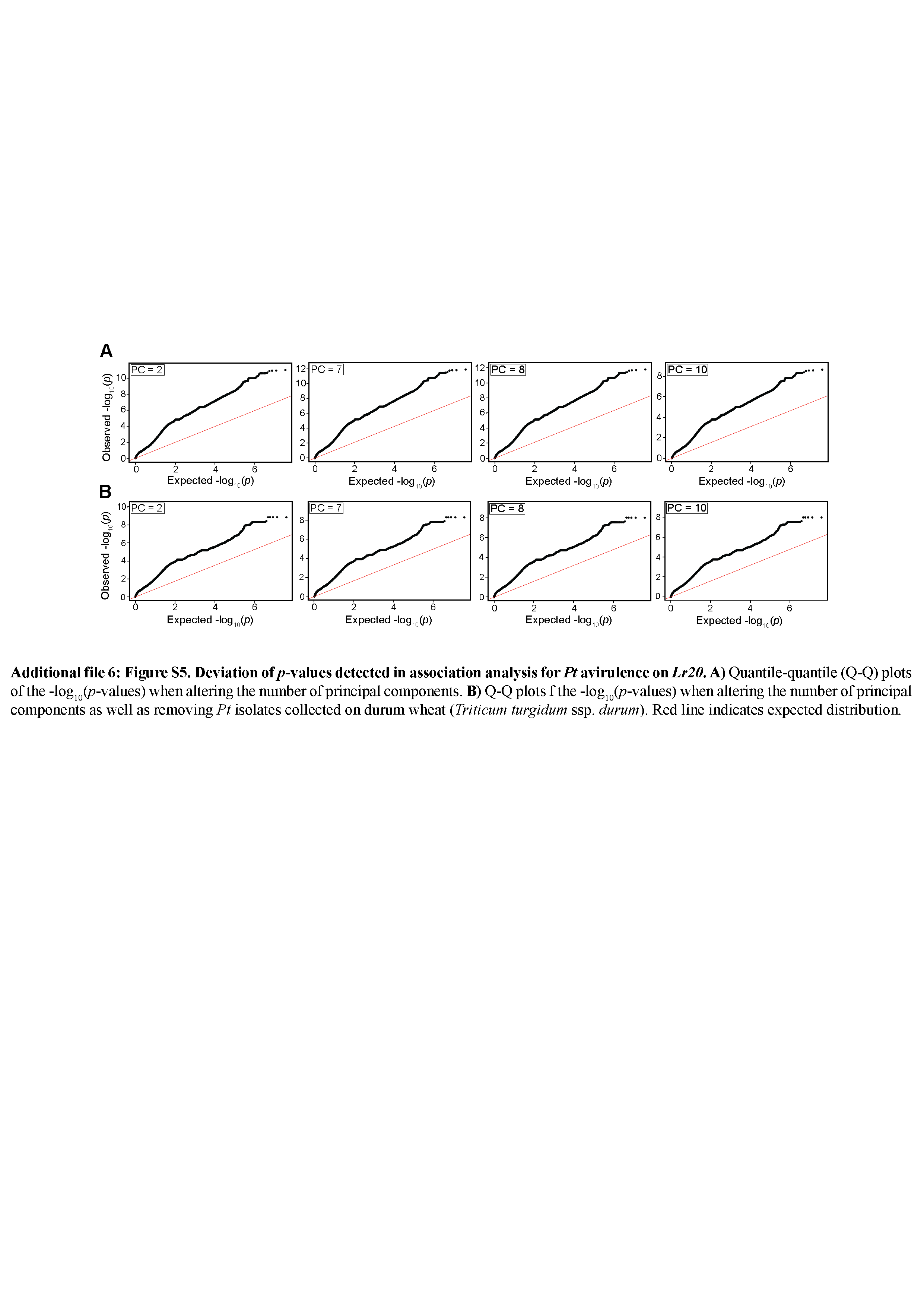

Supplement: Supplementary file 6 — Supplementary Material 6 [file 12864_2025_12230_MOESM6_ESM.tif]

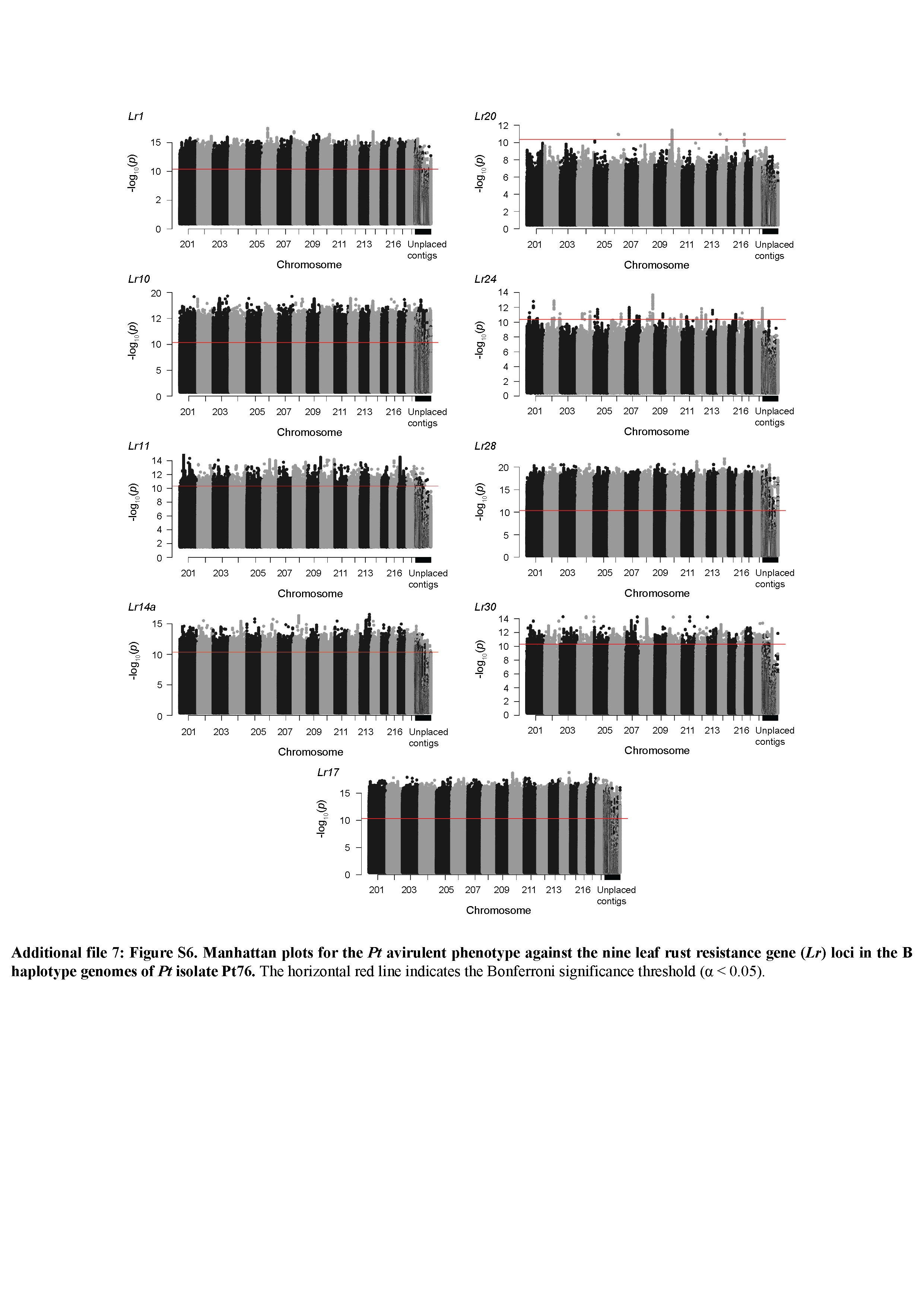

Supplement: Supplementary file 7 — Supplementary Material 7. [file 12864_2025_12230_MOESM7_ESM.tif]

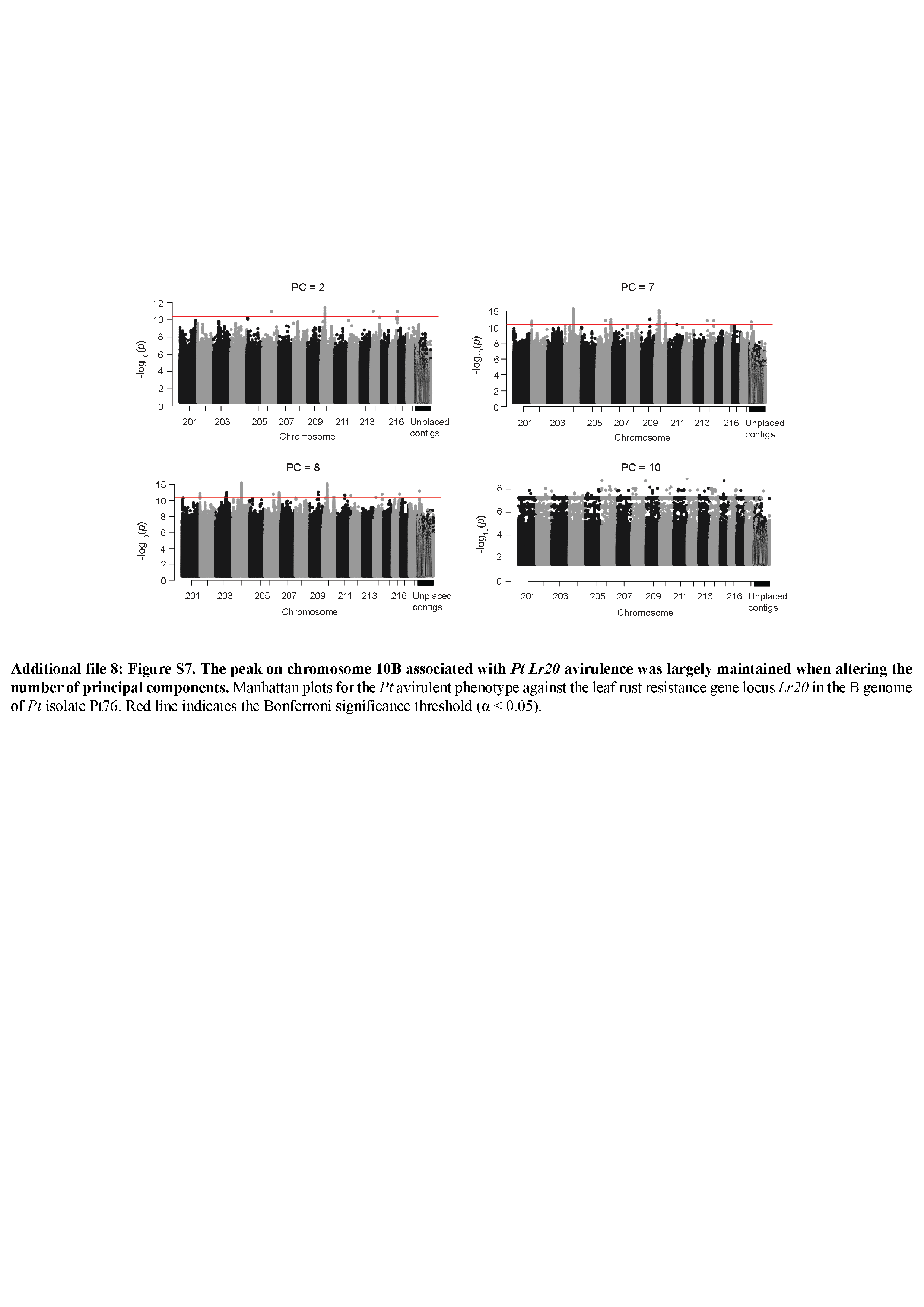

Supplement: Supplementary file 8 — Supplementary Material 8. [file 12864_2025_12230_MOESM8_ESM.tif]

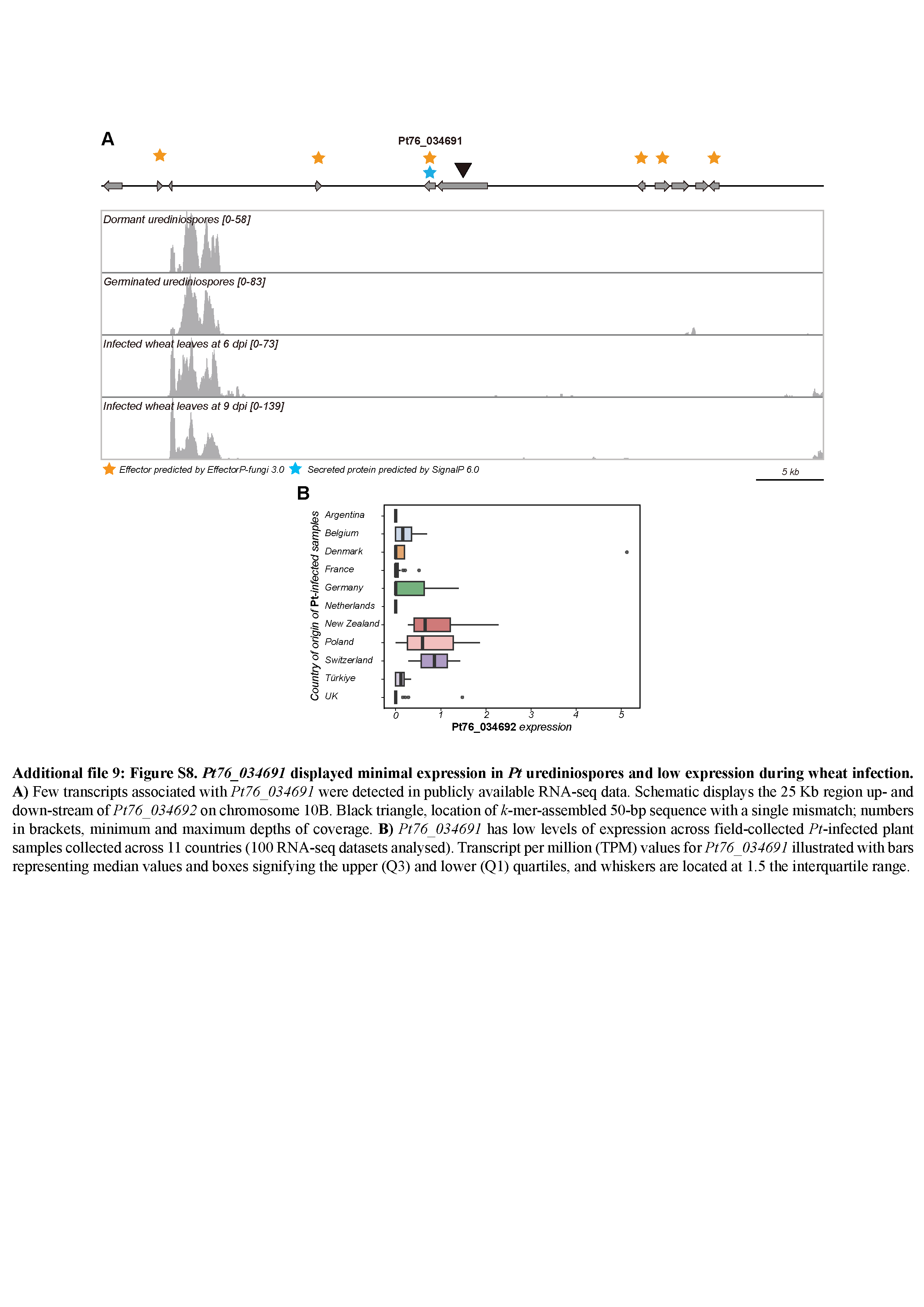

Supplement: Supplementary file 9 — Supplementary Material 9. [file 12864_2025_12230_MOESM9_ESM.tif]

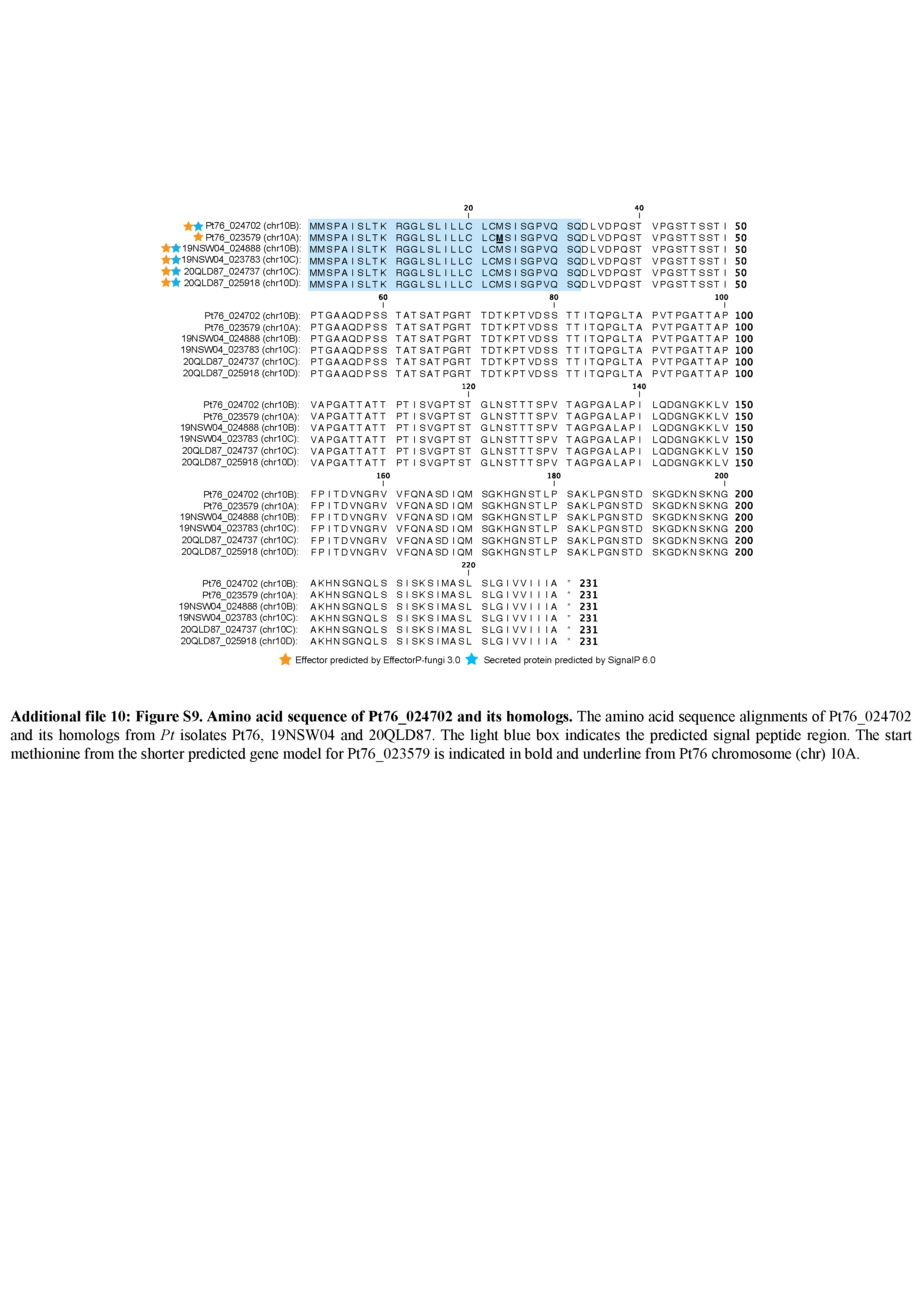

Supplement: Supplementary file 10 — Supplementary Material 10. [file 12864_2025_12230_MOESM10_ESM.tif]

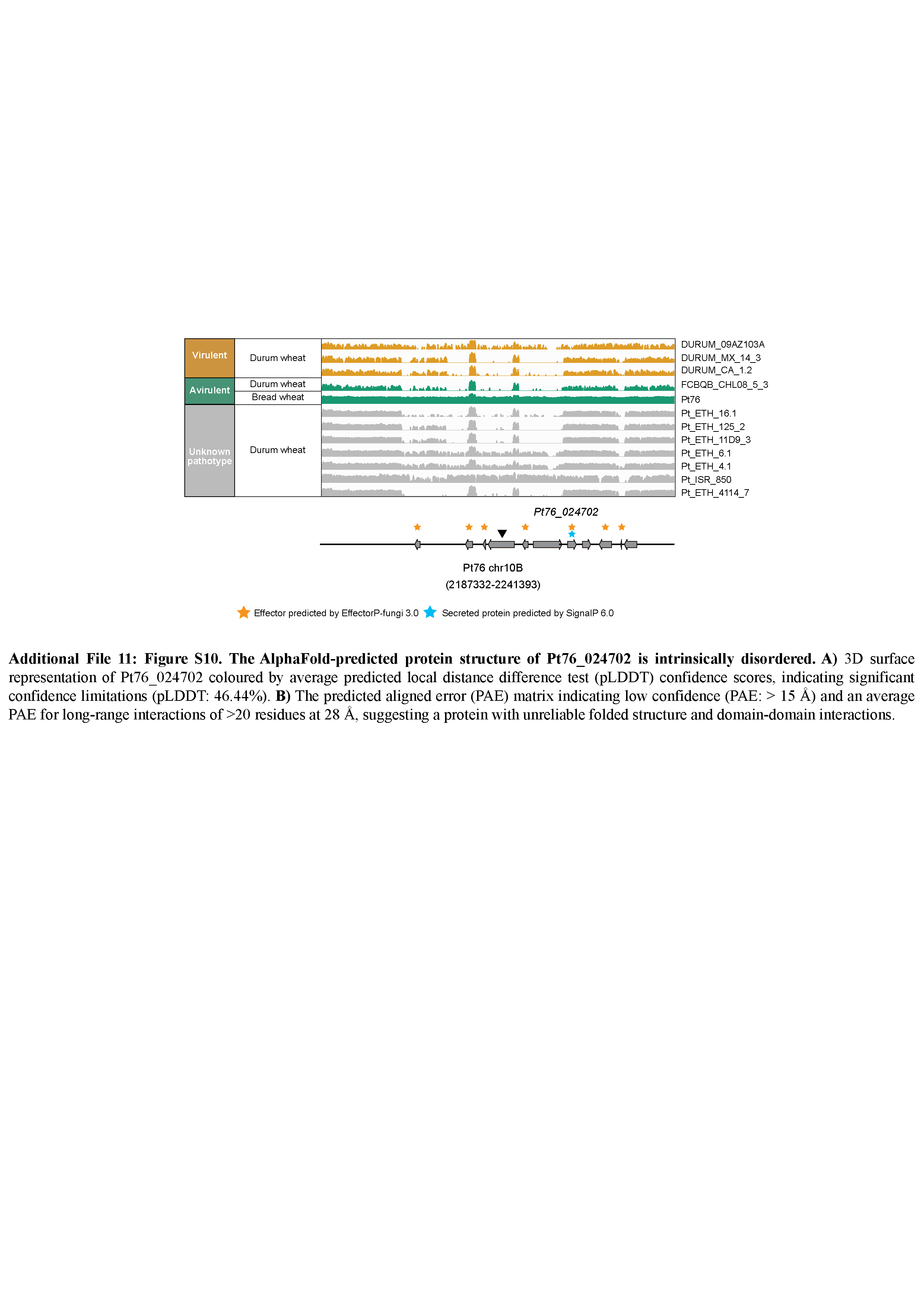

Supplement: Supplementary file 11 — Supplementary Material 11. [file 12864_2025_12230_MOESM11_ESM.tif]

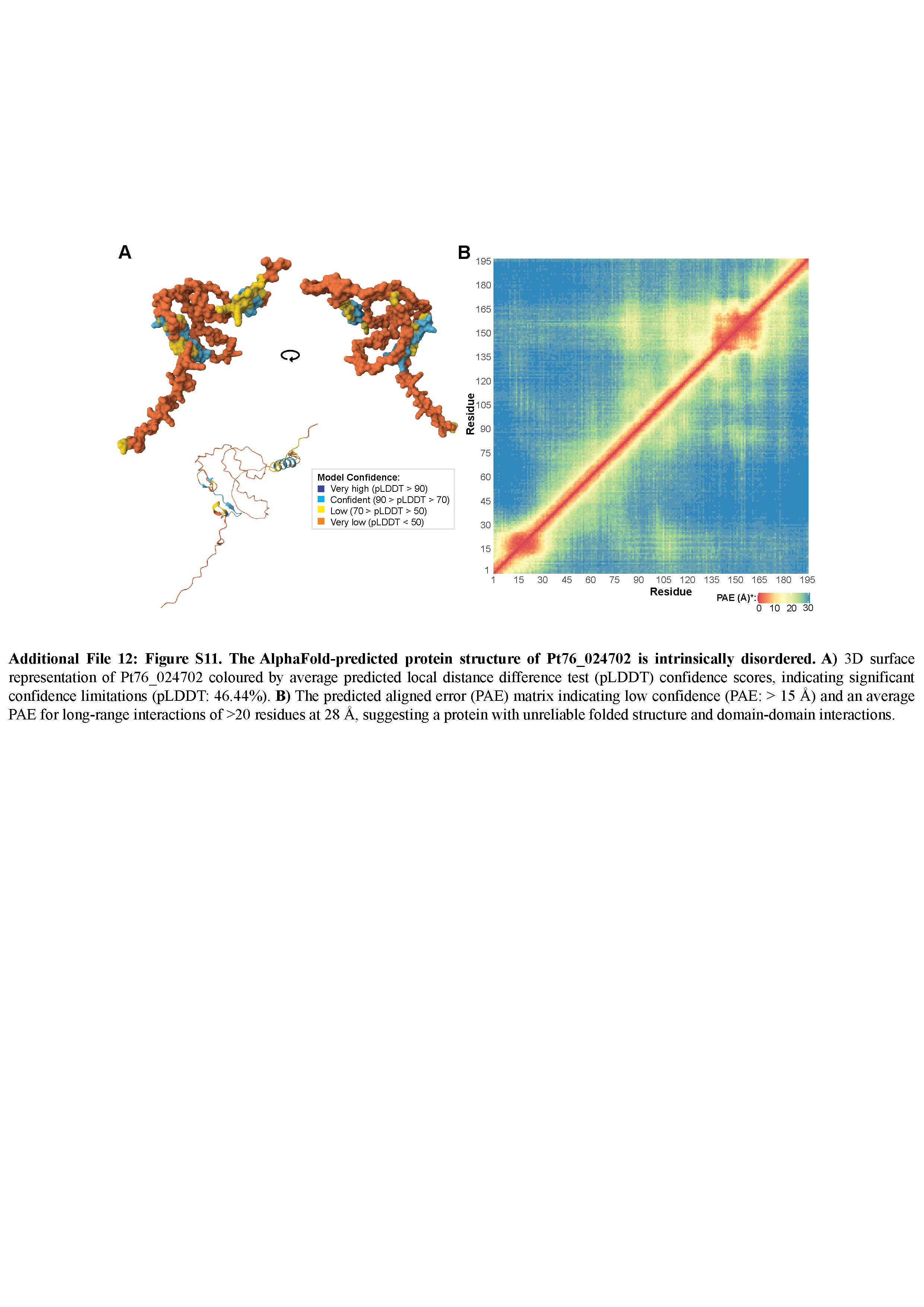

Supplement: Supplementary file 12 — Supplementary Material 12. [file 12864_2025_12230_MOESM12_ESM.tif]
